# Integrated re-analysis of transcriptomic and proteomic datasets reveals potential mechanisms for Zika viral-based oncolytic therapy in neuroblastoma

**DOI:** 10.12688/f1000research.132627.2

**Published:** 2023-11-20

**Authors:** Matt Sherwood, Yilu Zhou, Yi Sui, Yihua Wang, Paul Skipp, Carolini Kaid, Juliet Gray, Keith Okamoto, Rob M. Ewing

**Affiliations:** 1 School of Biological Sciences, Faculty of Environmental and Life Sciences, University of Southampton, Southampton, England, SO17 1BJ, UK; 2 Human Genome and Stem-Cell Center (HUG-CELL), Biosciences Institute, Universidade de Sao Paulo, São Paulo, State of São Paulo, Brazil; 3 Centre for Cancer Immunology, Faculty of Medicine, University of Southampton, Southampton, England, UK

**Keywords:** Neuroblastoma, oncolytic virotherapy, Zika virus, transcriptomics, proteomics

## Abstract

**Background:**

Paediatric neuroblastoma and brain tumours account for a third of all childhood cancer-related mortality. High-risk neuroblastoma is highly aggressive and survival is poor despite intensive multi-modal therapies with significant toxicity. Novel therapies are desperately needed. The Zika virus (ZIKV) can access the nervous system and there is growing interest in employing ZIKV as a potential therapy against paediatric nervous system tumours, including neuroblastoma.

**Methods:**

Here, we perform extensive data mining, integration and re-analysis of ZIKV infection datasets to highlight molecular mechanisms that may govern the oncolytic response in neuroblastoma cells. We collate infection data of multiple neuroblastoma cell lines by different ZIKV strains from a body of published literature to inform the susceptibility of neuroblastoma to the ZIKV oncolytic response. Integrating published transcriptomics, interaction proteomics, dependency factor and compound datasets we propose the involvement of multiple host systems during ZIKV infection.

**Results:**

Through data mining of published literature, we observed most paediatric neuroblastoma cell lines to be highly susceptible to ZIKV infection and propose the PRVABC59 ZIKV strain to be the most promising candidate for neuroblastoma oncolytic virotherapy. ZIKV induces TNF signalling, lipid metabolism, the Unfolded Protein Response (UPR), and downregulates cell cycle and DNA replication processes. ZIKV infection is dependent on sterol regulatory element binding protein (SREBP)-regulated lipid metabolism and three protein complexes; V-ATPase, ER Membrane Protein Complex (EMC) and mammalian translocon. We propose ZIKV non-structural protein 4B (NS4B) as a likely mediator of ZIKVs interaction with IRE1-mediated UPR, lipid metabolism and mammalian translocon.

**Conclusions:**

Our work provides a significant understanding of ZIKV infection in neuroblastoma cells, which will facilitate the progression of ZIKV-based oncolytic virotherapy through pre-clinical research and clinical trials.

## Keypoints

• The Zika virus may provide the basis for an oncolytic virotherapy against Neuroblastoma
• Most paediatric neuroblastoma cell lines are susceptible to Zika viral infection
• We highlight molecular mechanisms that may contribute to the oncolytic response in Neuroblastoma

## Contribution to the field

The ability to both induce direct oncolysis and provoke an anti-tumoral immune response makes oncolytic virotherapy an attractive candidate to combat aggressive and heterogenous cancers, such as high-risk neuroblastoma. To progress oncolytic virotherapy to clinical trial it is essential to understand the host mechanisms the virus manipulates to kill cancer cells, alongside any pathology as a consequence of infection of normal cells. Through data mining and re-analysing published data we observed that ZIKV efficiently infects and induces oncolysis of paediatric neuroblastoma cells and we propose a potential TNF pathway-driven immune response. ZIKV’s specificity for infection of nervous system cancer cells, while rarely causing nervous system-related pathology in young children, addresses many of its safety concerns. The inclusion of more effective and less toxic novel therapies, such as a potential ZIKV-based therapeutic, in multimodal treatment regimens will pave the way for improving patient long-term health and overall survival.

## Introduction

Neuroblastoma is the most common extracranial solid cancer in children, accounting for 6–10% of all paediatric cancers and disproportionately causing 12–15% of paediatric cancer-related deaths. ^1^ It is an embryonal tumour originating from transformed cells of neural crest lineage and predominately forms tumours in the adrenal medulla and paraspinal sympathetic ganglia. Whilst the majority of patients are diagnosed by the age of 5 years, the median age of patients is 18 months. Prognosis is highly heterogenous and can be predicted by a number of factors, including the presence of metastatic disease, age, chromosomal aberrations and molecular signatures, such as MYC-N amplification. ^2^ Patients are categorised according to internationally agreed risk groups (INRG), and treatment is stratified accordingly.

Outcome for low- and intermediate-risk neuroblastoma is good, with some patients requiring little or no treatment. However, approximately 50% of patients have high-risk disease, for which prognosis is poor, with overall survival of less than 60%. ^3^ Current high-risk neuroblastoma treatment regimens are aggressive. These include multiple rounds of induction chemotherapy, surgical resection, myeloablative chemotherapy, autologous stem cell transplantation and post-consolidation therapy such as immunotherapy. ^4^ The aggressive nature of this regimen carries significant treatment-related mortality and frequently results in long-term toxicities and sequelae impacting the quality of life for surviving patients. Consequently, there is a clear and unmet need for safer and less toxic treatment regimens to combat high-risk neuroblastoma.

Oncolytic virotherapy exploits viruses that preferentially infect and destroy cancer cells *via* two distinct routes of therapeutic action. Following infection, intense viral replication induces oncolysis, releasing virions into the tumour microenvironment to infect neighbouring tumour cells. Induction of a tumour-specific immune response is a crucial secondary mechanism employed by oncolytic virotherapy that can address highly heterogeneous tumours such as high-risk neuroblastoma and central nervous system (CNS) tumours. There is significant interest in combining immuno-modulating cancer therapies with oncolytic virotherapy to augment the anti-tumoral immune response. Oncolytic virotherapy clinical studies have in general reported low toxicity and minimal adverse effects in patients, mainly low-grade constitutional symptoms. ^5^

Zika virus (ZIKV) is a mosquito-borne flavivirus consisting of historical African and epidemic-associated Asian lineages. The latter can access the central nervous system and may cause microcephaly in the developing foetus through infection of neural stem and progenitor cells, causing cell death and growth reduction. ^6^^,^^7^ By contrast, ZIKV rarely causes adverse effects in children and adults, with the majority of cases (50–80%) being asymptomatic. ^8^ In symptomatic children, ZIKV may cause short-term side effects, namely rash, fever and gastrointestinal symptoms, and in rare instances in adults can cause more severe conditions, such as Guillain-Barré Syndrome, meningitis and encephalitis. ^8^^,^^9^

Since 2017, the concept of employing the ZIKV as oncolytic virotherapy against brain tumours has gained momentum. ZIKV induces an oncolytic event in infected paediatric brain tumour cells *in vitro* and *in vivo* assays and induces an immune response against spontaneous canine brain tumours. ^10^^–^^12^ An initial study assessing ZIKV infection in multiple neuroblastoma cell lines demonstrated ZIKV’s potential as a novel neuroblastoma oncolytic virotherapy. ^13^ Here, we survey over 35 studies that have used neuroblastoma cell lines to model ZIKV infection. These studies focused on understanding ZIKV pathology and assessing anti-viral compounds. Through re-analysis and integration of the transcriptomics, proteomics and dependency factor screens from these studies, we propose multiple molecular mechanisms to be implicated in ZIKV infection of neuroblastoma which help to determine its potential as oncolytic virotherapy.

## Methods

### RNA-Seq data source and processing

RNA-Seq data files (.fastq.gz paired-end) were acquired from the European Nucleotide Archive (ENA) (accession: PRJNA630088). Bonenfant et al., generated this data by infecting SH-SY5Y cells in monolayer culture with ZIKV PRVABC59 at MOI 5 for 1 hour and collected RNA at 24hpi vial TRIzol extraction. ^14^ RNA-seq libraries were prepared and sequenced using an Illumina NextSeq500. Our RNA-Seq processing pipeline consisted of FastQC (V0.11.9-0) (RRID:SCR_014583), ^15^^,^^16^ Trim Galore (V0.6.6-0) (RRID:SCR_011847), HISAT2 (V2.2.0) (RRID:SCR_015530), ^17^ SAMTOOLS (V1.11) (RRID:SCR_002105) ^18^ and Subread (V2.0.1) (RRID:SCR_009803). ^19^ Reads were aligned against the *Homo sapiens* GRCh38 genome.

### RNA-Seq differential gene expression and pathway analysis

Differential gene expression analysis was performed using DESeq2 (RRID:SCR_015687) ^20^ to compare the ZIKV-infected SH-SY5Y cells *versus* the non-infected control cells at 24hpi (n = 3). Differentially expressed genes (DEGs) were plotted on bar charts, volcano and scatter plots using GraphPad Prism (9.2.0) (RRID:SCR_002798). DEGs (padj < 0.05, fold change > 1.5) were submitted to Database for Annotation, Visualization and Integrated Discovery (DAVID) (DAVID 2021 (Dec. 2021)) (RRID:SCR_001881) ^21^^,^^22^ as official gene symbols for Gene Ontology (GO) (RRID:SCR_002811) (Biological Process Direct), ^23^^,^^24^ Kyoto Encyclopedia of Genes and Genomes (KEGG) (RRID:SCR_012773) ^25^^–^^27^ and Reactome (RRID:SCR_003485) pathway analysis. ZIKV-induced DEGs were mapped onto Pathview (RRID:SCR_002732). ^28^ Significance values of DEG and pathway analysis were corrected for multiple testing using the Benjamini and Hochberg method (padj < 0.05).

### ZIKV interactome source and analysis

We sourced 130 and five high-confidence interactions of ZIKV NS4B and NS2B-3, respectively, in SK-N-BE2 paediatric neuroblastoma cells from IMEx - The International Molecular Exchange Consortium (RRID:SCR_002805) (IM-26452). ^29^ Scaturro et al., produced this interactome through stable expression of HA-tagged ZIKV proteins in SK-N-BE2 cells, isolation of ZIKV-host protein complexes through HA-affinity purifications, then sample preparation and run for LC–MS/MS (n = 4). ^30^ Raw data was processed using MaxQuant with Andromeda search engine. High-confidence interactions were determined by Bayesian statistical modelling, with log2(fold change) ≥ 2.5; unadjusted one-sided P ≤ 0.05.

The viral-host interactome was submitted to STRING (RRID:SCR_005223) ^31^ for high confidence (0.7) evidence-based physical subnetwork analysis to identify host-host interactions. We integrated and mapped the viral-host and STRING derived host-host interactions in Cytoscape (3.9.1) (RRID:SCR_003032) ^32^ to identify the interaction of ZIKV NS4B with host protein complexes. ZIKV NS4B host interaction partners were also submitted to DAVID to identify the interaction between NS4B and host pathways. ZIKV dependency factors were integrated into Cytoscape map to highlight known protein interaction of ZIKV dependency factors with ZIKV proteins.

### ZIKV dependency factor source and analysis

Extensive data mining revealed there are currently only 22 known ZIKV dependency factors in neuroblastoma cells, identified via a shRNA screen in paediatric SK-N-BE2 neuroblastoma cells. ^30^ In an attempt to supplement this limited pool of ZIKV dependency factors, we sourced known factors from genome-wide CRISPR/Cas9 screens performed in glioma stem cells (GSCs), ^33^ hiPSC-NPC, ^34^ HEK293FT ^33^ and HeLa cells. ^35^ All can ZIKV dependency factors are shown in Table 4. A combination of DAVID GO and pathway analysis, STRING interaction and literature mining approaches were employed to identify relationships between host factors to inform dependencies of ZIKV on host protein complexes and cell machinery.

## Results and Discussion

### ZIKV displays strong oncolytic properties against neuroblastoma cells

ZIKV infects and significantly reduces the cell viability of a multitude of neuroblastoma cell lines from both primary tumour and metastatic sites ( Table 1). ZIKV can significantly reduce neuroblastoma cell viability at multiplicity of infection (MOI) as low as 0.001. ^36^ The cell viability of 11/15 neuroblastoma cell lines is significantly reduced to approximately 20% or less following ZIKV infection and these observations are apparent despite the differences in the cell line, ZIKV strain, viral MOI and the type of assay performed ( Table 1). SK-N-BE1 and SK-N-BE2 cells are from bone marrow metastasis from the same patient before and after treatment, respectively, and are both highly susceptible to ZIKV. SK-N-AS, T-268 and JFEN are highly resistant (cell viability >80%) to ZIKV infection. Susceptibility is independent of patient sex, cell line origin, morphology and MYC-N status ( Table 1). The non-sympathetic nervous system and non-paediatric origin of the T-268 and JFEN cells likely explain their resistance to ZIKV infection, as ZIKV has a tropism for paediatric nervous system cancer cells. ^11^ The resistance of the paediatric SK-N-AS cell line is governed by CD24 expression, which regulates the basal antiviral state of these cells. ^13^^,^^37^ Whilst LA-N-6 shows partial resistance to ZIKV infection ( Table 1), analysis of bulk mRNA and protein show cell LA-N-6 to express CD24. ^13^ Potential reasoning for this partial resistance is that subpopulations within LA-N-6 may be CD24 ^-^ or a CD24-independent mechanism may be employed to infer resistance. From Table 1 we conclude ZIKV to be a promising oncolytic virotherapy candidate to employ against paediatric neuroblastoma since it can target neuroblastoma cells originating from the primary tumour, metastatic sites, and metastatic sites that are resistant to standard neuroblastoma therapy.

**Table 1.** ZIKV infects and reduces cell viability in a multitude of paediatric neuroblastoma cell lines. Cell lines are ranked by the degree to which ZIKV infection reduces their cell viability. Cell viability assay reagent/marker used in the original assay is stated with its accompanying reference. *Note:* ZIKV, Zika virus; MYCN, MYCN Proto-Oncogene; NA, not applicable.

**Cell line**	**Cell viability**	**Viability Reagent/Marker**	**Patient Age/Sex**	**Cancer type**	**Cell line origin**	**Morphology**	**MYCN status**
SH-SY5Y	<20%	Annexin V, ^63^ Annexin V/7-AAD, ^68^ Eosin-Y ^69^^,^^70^	4/F	Neuroblastoma	Bone marrow metastasis (thorax)	Epithelial	non-amplified
SK-N-SH	<20%	Reliablue, ^39^ Giemsa ^71^	4/F	Neuroblastoma	Bone marrow metastasis (thorax)	Epithelial	non-amplified
SK-N-BE2	<20%	Eosin-Y ^69^^,^^70^	2/M	Neuroblastoma	Bone marrow metastasis	Neuroblast	amplified
SK-N-BE2-M17	<20%	Eosin-Y ^70^	2/M	Neuroblastoma	Bone marrow metastasis	Neuroblast	amplified
SK-N-DZ	<20%	Eosin-Y ^69^^,^^70^	2/F	Neuroblastoma	Bone marrow metastasis	Epithelial	amplified
IMR-32	<20%	MTS, ^13^ Eosin-Y ^69^^,^^70^	1/M	Neuroblastoma	Abdominal mass primary tumour	Neuroblast, Fibroblast	amplified
SMS-KAN	<20%	MTS ^13^	3/F	Neuroblastoma	Pelvic primary tumour	Neuroblast	amplified
SMS-KCNR	<20%	Eosin-Y ^69^	1/M	Neuroblastoma	Bone marrow metastasis (adrenal)	Neuroblast	amplified
SK-N-FI	<20%	Eosin-Y ^69^	11/M	Neuroblastoma	Bone marrow metastasis	Epithelial	non-amplified
CHLA-42	<20%	MTS ^13^	1/NA	Neuroblastoma	Bone marrow metastasis	Epithelial	non-amplified
SK-N-BE1	~20%	MTS ^13^	2/M	Neuroblastoma	Bone marrow metastasis	Neuroblast	amplified
LA-N-6	~60%	MTS ^13^	5/M	Neuroblastoma	Bone marrow metastasis (adrenal)	Neuroblast	non-amplified
SK-N-AS	>80%	MTS ^13^	6/F	Neuroblastoma	Bone marrow metastasis (adrenal)	Epithelial	non-amplified
T-268	>80%	Eosin-Y ^69^	22/F	Olfactory neuroblastoma	Metastasis (paraspinal mass)	NA	NA
JFEN	>80%	Eosin-Y ^69^	22/M	Olfactory neuroblastoma	Metastasis (chest wall)	NA	NA

### ZIKV strains possess different therapeutic potential against neuroblastoma cells

Independent studies have demonstrated inherent differences in the ability of varying ZIKV strains to infect, replicate, and kill neuroblastoma cells. ^38^^,^^39^ Here, we assess published data concerning ZIKV infection of neuroblastoma cells and ranked the viral strains based on their ability to infect neuroblastoma cells, produce fresh viral progeny and reduce cell viability ( Table 2). Data mining showed the PRVABC59 Asian and Uganda #976 African strains as the top two candidates ( Table 2). The PRVABC59 Asian strain induces significantly more DEGs and splice events of immune and inflammatory response genes in SH-SY5Y cells compared to the African MR766 strain, which has 99.95% sequence identity to Uganda #976. ^14^ Brain metastases develop in 5–11% of patients with neuroblastoma and are correlated with poor prognosis. ^40^ The ability of the Asian lineage to access the brain may enhance the therapeutic potential of ZIKV by targeting these brain metastases. Population level data has shown the epidemic Asian ZIKV lineage to rarely cause anything other than mild symptoms in children and adolescents, thus providing evidence for the safety of employing an Asian strain. ^41^ Consequently, from those tested to date, we propose that the PRVABC59 Asain strain holds the greatest promise for development as oncolytic virotherapy against paediatric neuroblastoma.

**Table 2.** Different ZIKV strains demonstrate varying therapeutic potential against paediatric neuroblastoma cells. ZIKV strains are ranked by their ability to infect (Degree of Infection), replicate within (Viral Titer) and significantly reduce the cell viability (Cell Viability) of a multitude of neuroblastoma cells. The Data accordance is a qualitative measure which we employed to describe the degree of similarity of the results between publications that performed ZIKV infection assays of neuroblastoma cells using the same ZIKV strain. Data accordance of five denotes that the findings of one publication closely support the findings from another, a data accordance of one denotes publications reporting vastly contrasting results. When a viral strain is published in only one paper, it is allocated a data accordance of NA. ZIKV, Zika virus; NA, not applicable.

**ZIKV strain**	**Lineage**	**Cell Viability**	**Viability Reagent/Marker**	**Degree of infection**	**Viral titer**	**MOI Range**	**Data accordance**	**Infection Conditions**
**PRVABC59**	Asian	<20%	MTS ^13^, Eosin-Y ^69^	>80%	>10 ^7^ per ml	0.5-10	5	All cell lines in Table 1, excluding SK-N-BE2-M17. ^13^^,^^69^^,^^72^
**Uganda #976**	African	<20%	Reliablue ^39^	>60%	10 ^6^-10 ^7^ per ml	0.01-10	4	SH-SY5Y ^51^, SK-N-SH ^38^^,^^39^
**Brazil PE/243**	Asian	20-40%	MTT ^46^, Annexin V/7-AAD ^68^	>60%	>10 ^7^ per ml	0.5-10	4	SH-SY5Y ^46^^,^^68^^,^^73^
**MR766**	African	20-40%	Annexin V ^63^, Giemsa ^71^	>60%	>10 ^7^ per ml	0.01-10	3	SH-SY5Y ^56^^,^^63^^,^^74^^,^^75^, SK-N-SH ^38^^,^^71^^,^^72^^,^^76^
**SZ01/2016/China**	Asian	<20%	Annexin V ^63^	NA	>10 ^7^ per ml	1	NA	SH-SY5Y ^63^
**French Polynesia/2013**	Asian	20-40%	Reliablue ^39^	>20%	10 ^6^-10 ^7^ per ml	0.01-10	4	SH-SY5Y ^51^^,^^77^, SK-N-SH ^38^^,^^39^
**HS-2015-BA-01**	Asian	40-60%	WST-1 ^78^	NA	>10 ^7^ per ml	0.01-1	4	SH-SY5Y ^36^^,^^78^
**Paraiba/2015**	Asian	20-40%	Giemsa ^71^	NA	10 ^6^-10 ^7^ per ml	0.1-10	2	SK-N-SH ^71^^,^^79^
**BR/800/16 Brazil 2016**	Asian	40-60%	Reliablue ^39^	>20%	10 ^6^-10 ^7^ per ml	0.1-1	NA	SK-N-SH ^39^
**PLCal_ZV**	Asian	>80%	WST-1 ^78^	NA	<10 ^4^ per ml	0.01-1	NA	SH-SY5Y ^78^

### ZIKV infection of neuroblastoma cells induces changes at the transcriptome level

**Figure 1.**
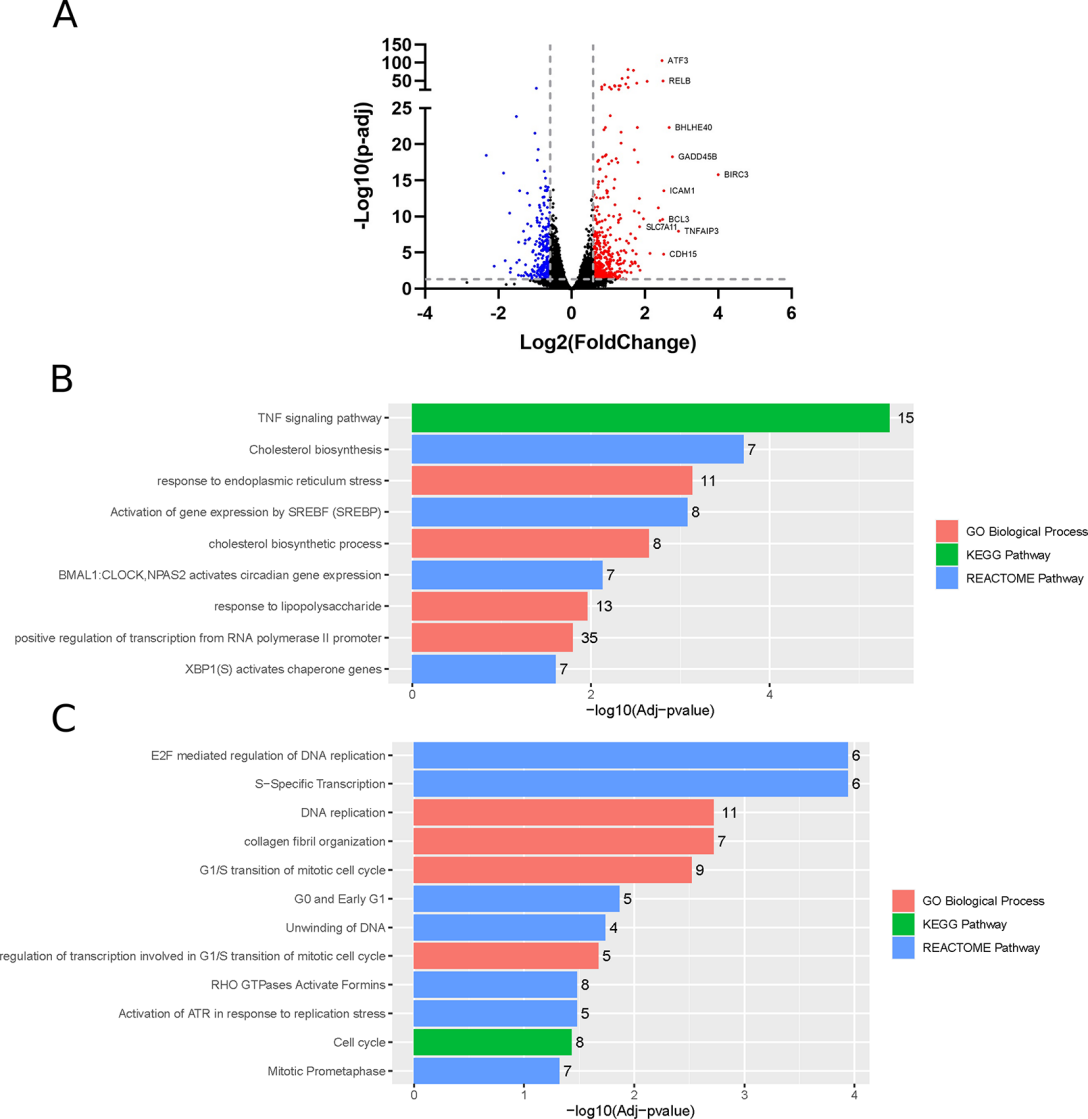
Differential gene expression, GO and pathway analysis of ZIKV infection in SH-SY5Y cells. Volcano plot of genes differentially expressed in response to ZIKV infection of SH-SY5Y cells, with the top 10 upregulated genes labelled (A). Significantly upregulated (B) and downregulated (C) GO Biological Processes, KEGG and Reactome pathways in response to ZIKV infection in SH-SY5Y neuroblastoma cells. Significance values are corrected for multiple testing using the Benjamini and Hochberg method (padj < 0.05). GO, Gene Ontology; ZIKV, Zika virus; DEG, differentially expressed gene.

**Figure 2.**
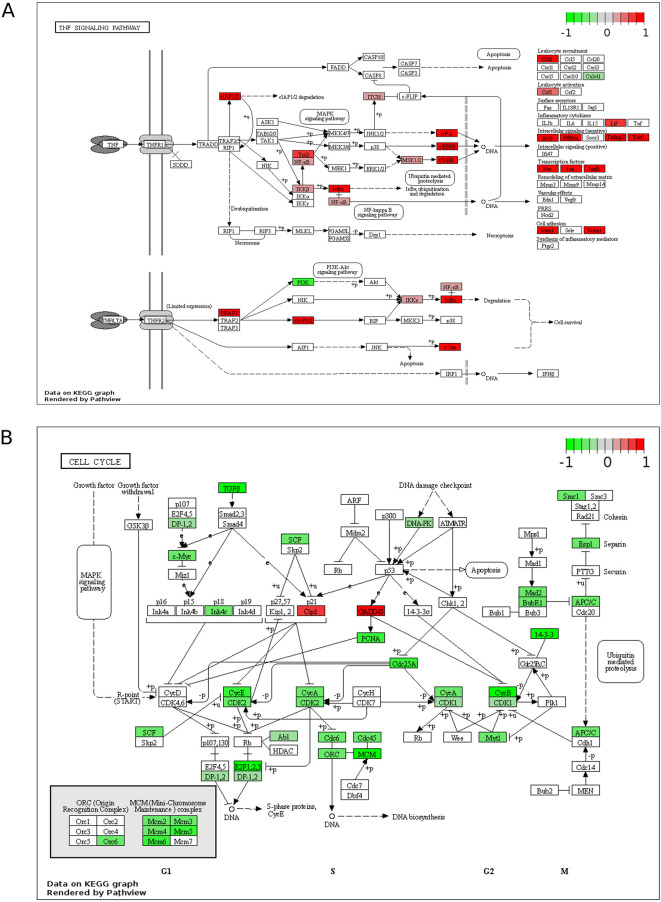
Pathway mapping of ZIKV infection in SH-SY5Y cells. KEGG maps of the up-reulated TNF Signalling Pathway (A) and down-regulated Cell Cycle (B), plotted using all DEGs (fold change > 0). Significance values are corrected for multiple testing using the Benjamini and Hochberg method (padj < 0.05). ZIKV, Zika virus; KEGG, Kyoto Encyclopedia of Genes and Genomes; DEG, differentially expressed gene.

Differential gene expression analysis identifies 453 and 256 significantly upregulated and downregulated genes (fold change > 1.5), respectively, in ZIKV-infected paediatric neuroblastoma SH-SY5Y cells ( Figure 1A). GO, Reactome and KEGG pathway analysis identifies nine significantly upregulated and 12 significantly downregulated terms ( Figure 1B- C). Upregulated processes include “TNF signalling pathway”, lipid metabolism (“Cholesterol biosynthesis”, “Cholesterol biosynthetic process”, “Activation of gene expression by SREBF (SREBP)”), endoplasmic reticulum (ER) stress (“Response to endoplasmic reticulum stress”, “XBP1(S) activates chaperone genes”) and transcription (“BMAL1:CLOCK, NPAS2 activates circadian gene expression”, “Positive regulation of transcription from RNA polymerase II promoter”). The downregulated terms are predominantly cell cycle- and DNA replication-related processes and this downregulation is apparent when the “Cell Cycle” KEGG pathway is plotted for all DEGs (fold change > 0) ( Figure 2B). A potential explanation for this observation is that ZIKV can disrupt the cell cycle by targeting the centrioles in neuroblastoma cells. ^42^

### ZIKV induces TNF signalling in neuroblastoma cells

**Figure 3.**
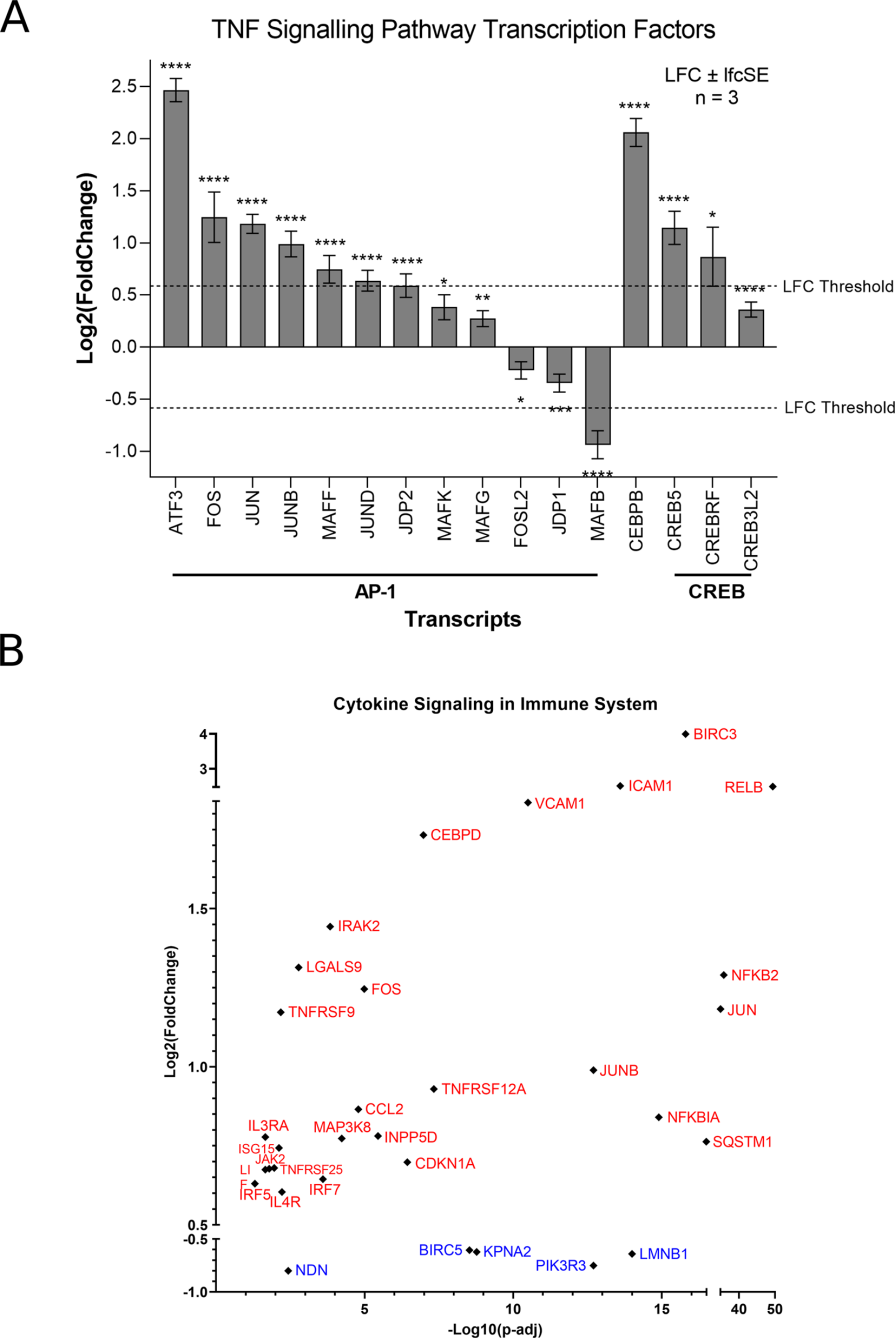
ZIKV infection upregulates the TNF signalling pathway in neuroblastoma cells. Expression levels of TNF Signalling Transcription Factors in SH-SY5Y cells in response to ZIKV infection (A). Expression levels of cytokine signalling in immune system genes in SH-SY5Y cells in response to ZIKV infection (B). Significance values are corrected for multiple testing using the Benjamini and Hochberg method (padj < 0.05). A threshold line of Log _2_(1.5 Fold Change) has been applied for the expression values. Log _2_FoldChange (LFC) ± standard error of the LFC estimate (lfcSE), n = 3. ZIKV, Zika virus; DEG, differentially expressed gene; AP-1, activator protein 1; CREB, cAMP response element-binding protein.

Of the top 10 upregulated DEGs in SH-SY5Y cells, four (BIRC3, TNFAIP3, ICAM1 and BCL3) are components of the TNF signalling pathway ( Figure 1A). The TNF pathway is particularly noteworthy to consider for oncolytic virotherapy since it may play a role in both oncolysis (direct cell death) and the anti-tumoral immune response. Here, mapping the “TNF Signalling” KEGG pathway for ZIKV-infected SH-SY5Y cannot deduce if ZIKV may activate CASP-mediated apoptosis or CASP-independent necroptosis ( Figure 2A). However, ZIKV-infected SH-SY5Y cells clearly show significant upregulation of transcription factors (AP-1, cEBPβ and CREB), leukocyte recruitment and activation (CCL2 and CSF1), intracellular signalling (BCL3, NFKBIA, TNFAIP3 and TRAF1) and cell adhesion genes (Icam1 and Vcam1) ( Figure 2A). ZIKV significantly upregulates the expression of multiple Activator protein 1 (AP-1) transcription factors, including members from all four AP-1 subfamilies (ATF, JUN, FOS and MAF) in SH-SY5Y cells ( Figure 3A). AP-1 can regulate the expression of a diverse set of genes in response to nutrients, cytokines, stress or pathogen infection, and is involved in innate and adaptive immunity, differentiation, proliferation, survival and apoptosis. ^43^ AP-1 transcription factors can regulate the immune response of tumours, and significant AP-1 upregulation by ZIKV infection potentially identifies AP-1 as a mechanism through which ZIKV could yield an anti-tumoral immune response against neuroblastoma *in vivo.* ^44^

Here, CCL2 (MCP-1) is significantly upregulated at the transcriptome level by ZIKV infection, and two independent studies have shown CCL2 to be secreted by ZIKV-infected SH-SY5Y cells. ^45^^,^^46^ CCL2 is a pro-inflammatory mediator that recruits leukocytes *via* chemotaxis to infiltrate tissues, including the CNS, to stimulate inflammation. ^47^ A non-neurotoxic herpes simplex virus (HSV)-based oncolytic virotherapy, engineered to express physiologically relevant levels of CCL2 (M010), significantly reduced Neuro-2a neuroblastoma growth in the flank of immune-competent mice and recruited CD4+ and CD8+ T-cells to infiltrate the tumour. ^47^ Additionally, CCL2 is secreted by ZIKV-infected cultured canine glioblastoma cells when in the presence of monocytes and is detected in serum and CSF samples of canines bearing spontaneous brain tumours following ZIKV infection. ^12^ We propose here that CCL2 may be capable of inducing an anti-tumoral immune response against paediatric neuroblastoma during ZIKV infection. Supporting the notion of a ZIKV-induced inflammatory response, 32 genes implicated in cytokine signalling in the immune system are significantly differentially expressed in ZIKV-infected SH-SY5Y cells: 27 upregulated and five downregulated ( Figure 3B).

### ZIKV induces lipid metabolism in neuroblastoma cells

ZIKV infection significantly upregulates lipid metabolism-related terms in SH-SY5Y cells; specifically, “Cholesterol biosynthesis” and “Activation of gene expression by SREBF (SREBP)” ( Figure 1B). Cholesterol and lipids are essential cellular components and there are complex systems that function to regulate their intracellular abundance and localisation. These systems include regulation of cholesterol biosynthesis by the sterol regulatory element binding protein (SREBP) pathway, intracellular cholesterol trafficking, and cholesterol efflux by the liver X receptor (LXR) pathway. Cholesterol and fatty acids are required for multiple stages of the flavivirus life cycle, including regulating viral entry, the formation of viral replication complexes in the ER membrane and viral egress. ^48^ ZIKV elevates lipogenesis and remodels the composition of the lipid classes in infected SK-N-SH cells. ^49^ Our data mining of published literature identified several approaches to regulate ZIKV infection of neuroblastoma cells through modification of intracellular lipid levels ( Table 3). These include supplementation with pathway regulators (PF-429242, fenofibrate, lovastatin, U18666A and LXR 623) or exogenous lipids (oleic acid, docosahexaenoic acid (DHA) and cholesterol).

**Table 3.** ZIKV infection in neuroblastoma cells can be regulated through modifying lipid abundance, composition and localisation. List of compounds that regulate lipid homeostasis and are capable of restricting or enhancing ZIKV infection in paediatric neuroblastoma cells. ZIKV, Zika virus; LXR, liver X receptor; SREBP, sterol regulatory element binding protein; DHA, docosahexaenoic acid.

**Compound**	**Cell line**	**Mechanism of action**	**Effect on ZIKV infection**	**References**
Bafilomycin A1 (V-ATPase inhibitor)	SH-SY5Y	Impairs acidification of endosomal-lysosomal compartments	Restrict	^51^
U18666A	SH-SY5Y	Cholesterol accumulation impairs late endosomes & lysosomes	Restrict	^51^
LXR 623 (LXR pathway agonist)	SK-N-SH	Induces cholesterol efflux	Restrict	^52^
PF-429242 (SREBP pathway inhibitor)	SK-N-SH	Reduces intracellular lipid levels	Restrict	^49^
Fenofibrate (SREBP pathway inhibitor)	SK-N-SH	Reduces intracellular lipid levels	Restrict	^49^
Lovastatin (SREBP pathway inhibitor)	SK-N-SH	Reduces intracellular lipid levels	Restrict	^49^
Oleic Acid	SK-N-SH	Increases lipid droplet abundance	Enhance	^49^
Cholesterol	SK-N-SH	Increases lipid droplet abundance	Restrict	^49^
DHA	SH-SY5Y	Anti-inflammatory and neuroprotective effects against ZIKV infection	Restrict	^46^

**Figure 4.**
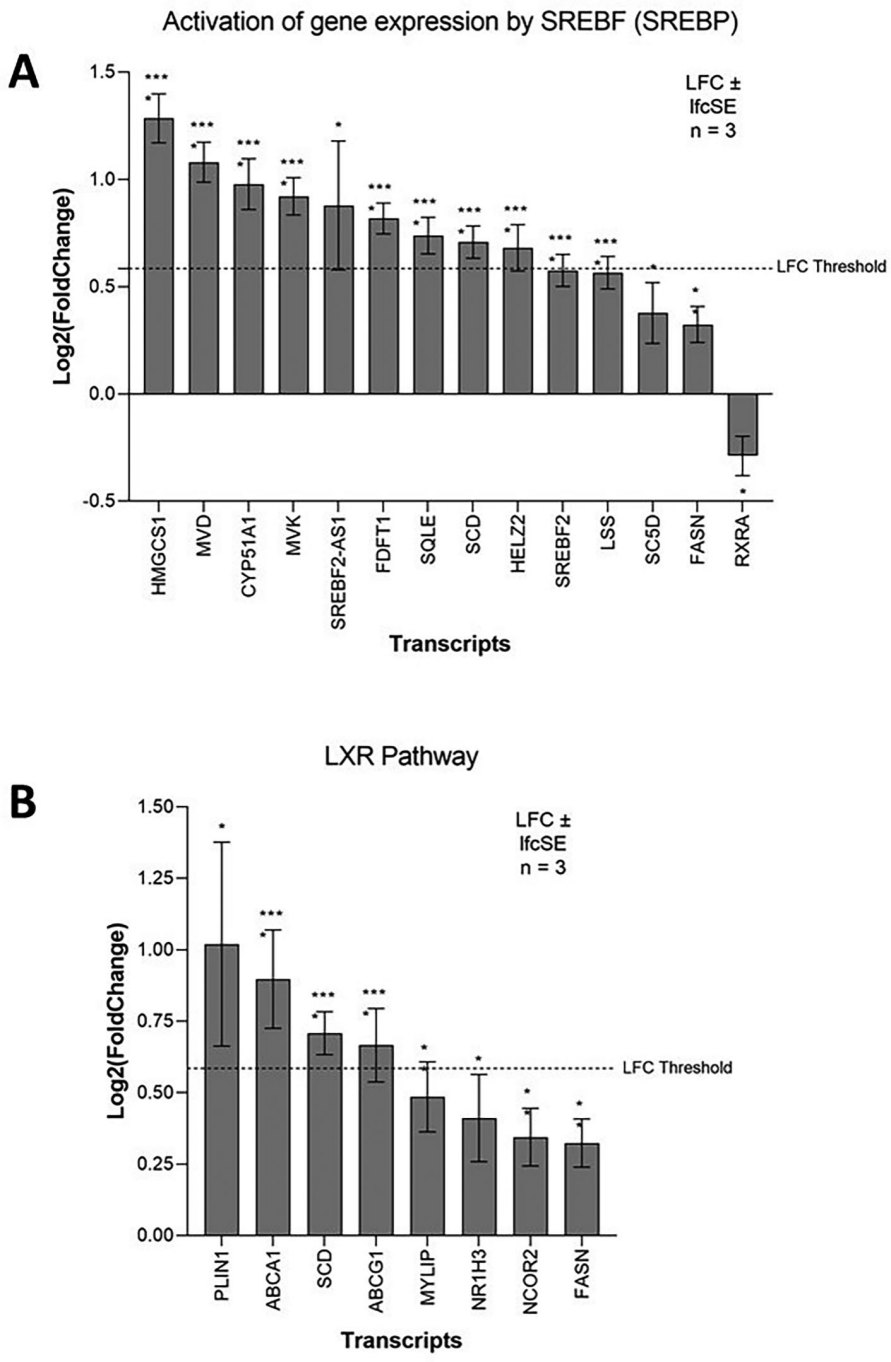
ZIKV infection upregulates lipid metabolism in neuroblastoma cells. Expression of SREBP pathway (A) and LXR pathway (B) genes in neuroblastoma cells in response to ZIKV infection. Significance values are corrected for multiple testing using the Benjamini and Hochberg method (padj < 0.05). A threshold line of Log _2_(1.5 Fold Change) has been applied for the expression values. Log _2_FoldChange (LFC) ± standard error of the LFC estimate (lfcSE), n = 3. ZIKV, Zika virus; SREBP, sterol regulatory element binding protein; LXR, liver X receptor.

Three SREBP pathway inhibitors (PF-429242, fenofibrate and lovastatin) reduce the capability of ZIKV to infect SK-N-SH neuroblastoma cells ( Table 3). The SREBP pathway is a principal regulator of fatty acid and cholesterol biosynthesis. The SREBF1 and SREBF2 transcription factors control this pathway, and although they share a small degree of redundancy, they primarily regulate the expression of fatty acid biosynthesis and cholesterol biosynthesis target genes, respectively. ^50^ Both SREBF2 and SREBF2-AS1 are significantly upregulated in ZIKV-infected SH-SY5Y cells, and the most highly upregulated SREBF downstream gene (HMGCS1) is a SREBF2 responsive gene ( Figure 4A). Pathway analysis identifies significant upregulation of “Cholesterol biosynthesis” ( Figure 1B) and here we observe significant upregulation of multiple enzymes of the SREBF2 cholesterol biosynthetic pathway (HMG-CoA synthase (HMGCS1), Mevalonate Diphosphate Decarboxylase (MVD), CYP51 (CYP51A1), Mevalonate Kinase (MVK), squalene synthase (FDFT1), Squalene Epoxidase (SQLE), Lanosterol Synthase (LSS), Lathosterol Oxidase (SC5D)) ( Figure 4A). The SREBP pathway is essential for ZIKV infection, and we propose that it may contribute, through the SREBF2 transcriptional pathway, to the upregulation of cholesterol biosynthesis in neuroblastoma cells.

Both U18666A and exogenous cholesterol restricts ZIKV infection of neuroblastoma cells ( Table 3). U18666A is an intracellular cholesterol transport inhibitor that causes the accumulation of cholesterol in lysosomes to hinder the endosomal-lysosomal system. ^51^ Exogenous cholesterol also leads to the inactivation of the late endosomal-lysosomal compartments through a build-up of cholesterol. ^51^ Collectively, this identifies a dependence of the ZIKV life cycle in neuroblastoma cells on intracellular cholesterol for the correct functioning of the endosomal-lysosomal system.

The LXR pathway agonist (LXR 623) promotes cholesterol efflux ( Table 3). Flaviviruses require cholesterol for the restructuring of host membranes, and LXR 623 demonstrates this dependence of ZIKV in neuroblastoma by preventing ZIKV-induced vesicle production and ER expansion in SK-N-SH cells. ^52^ The LXR pathway and expression of its downstream lipid homeostasis genes are regulated by the transcription factors LXR-α (NR1H3) and LXR-β (NR1H2). LXR-α protein is significantly increased by ZIKV infection of SK-N-SH neuroblastoma cells from 48 hr. ^52^ Although LXR-α mRNA is only marginally upregulated in our study, two major cholesterol efflux factors that are downstream of the LXR pathway, ATP-Binding Cassette A1 and G1 (ABCA1 and ABCG1), are significantly upregulated ( Figure 4B). Interestingly, exogenous addition of oleic acid enhances ZIKV infection in neuroblastoma cells ( Table 3). Oleic acid is one of the main monounsaturated fatty acid synthesised by the LXR pathway, which is achieved through induction of FASN and SCD. ^53^ We observe significant upregulation of both FASN and SCD in ZIKV-infected neuroblastoma cells ( Figure 4B) and propose that this may induce oleic acid synthesis to aid ZIKV infection in neuroblastoma cells. Collectively, our data mining identifies a dependence of ZIKV on the LXR pathway and suggests that ZIKV may manipulate this pathway in neuroblastoma cells to upregulate cholesterol efflux and/or induce oleic acid syntheis.

We propose that lipid abundance, localisation, trafficking and metabolism regulate ZIKV infection of neuroblastoma cells, and that remodelling of the cellular lipid composition within the host cell may produce a favourable environment for efficient replication.

### ZIKV induces and is dependent on the ER stress response in neuroblastoma cells

ZIKV upregulates ER-stress-related terms in SH-SY5Y cells, principally “Response to endoplasmic reticulum stress” and “XBP1(S) activates chaperone genes” ( Figure 1B). The Unfolded Protein Response (UPR) dictates the ER-stress response. The UPR is normally inactive due to the ER chaperone binding immunoglobulin protein (BIP) sequestering three ER stress sensors (IRE1, PERK and ATF6). Under stress conditions BIP releases IRE1, PERK and ATF6 to assist protein folding, allowing them to activate their respective UPR-mediated ER-stress pathways.

**Figure 5.**
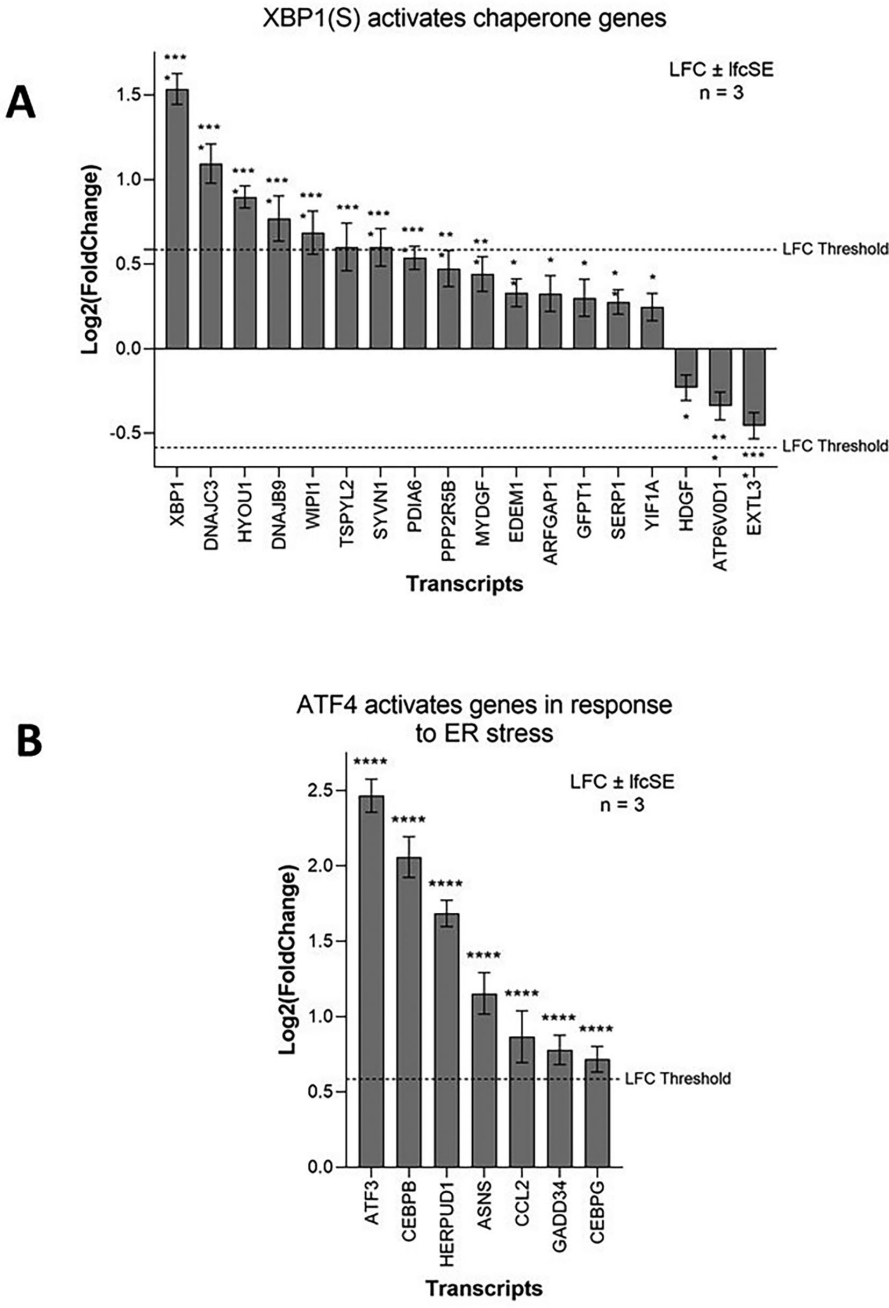
ZIKV infection activates the UPR in neuroblastoma cells. Expression levels of the XBP1(S) activates chaperone genes (A) and ATF4 activates genes in response to endoplasmic reticulum stress (B) genes in neuroblastoma cells in response to ZIKV infection. Significance values are corrected for multiple testing using the Benjamini and Hochberg method (padj < 0.05). A threshold line of Log _2_(1.5 Fold Change) has been applied for the expression values. Log _2_FoldChange (LFC) ± standard error of the LFC estimate (lfcSE), n = 3. ZIKV, Zika virus; UPR, Unfolded Protein Response; ER, endoplasmic reticulum.

Activation of the IRE1-mediated UPR leads to IRE1 splicing a 26 bp region from the ubiquitously expressed XBP1 mRNA. The active transcription factor XBP1(S) then drives the expression of genes to help alleviate ER stress, primarily chaperone and ER-associated protein degradation (ERAD) genes. ZIKV infection significantly upregulates 15 genes of the IRE1-mediated “XBP1(S) activates chaperone genes” Reactome pathway in SH-SY5Y cells ( Figure 5A). XBP1 is the most highly upregulated gene and others include the endoplasmic-reticulum-associated protein degradation (ERAD) gene SYVN1 and the chaperones DNAJC3 and DNAJB9 ( Figure 5A). Multiple IRE1-mediated UPR genes (EDEM1, SYVN1, SSR1, SRPRB, ATP6V0D1, and EXTL3) are ZIKV dependency factors in hiPSC-NPCs ( Table 4). Chemical inhibition of IRE1 by 4μ8C impairs ZIKV infection *in vivo.* ^54^ Our data mining and re-analysis shows that ZIKV significantly upregulates the IRE1-mediated UPR in SH-SY5Y cells and that ZIKV is dependent on this for efficient infection, likely as a means to regulate and combat viral replication-induced ER stress.

**Table 4.** ZIKV infection is dependent on multiple host factors. Lists of known ZIKV dependency factors across SH-SY5Y (NB), GSC, hiPSC-NPC (NPC), HEK293FT and HeLa cells. ZIKV, Zika virus; GSC, glioma stem cell; NB, neuroblastoma.

SH-SY5Y	GSC		hiPSC-NPC		HEK293FT		HeLa	
ATP6V0C	AKR1B10	MORF4L1	ANKAR	FBXL21	ALDH1L2	NR6A1	ABI2	MAPKAPK3
BSG	AKT1	MROH2B	AP2B1	FBXO45	AQP5	ORC4	AGAP1	MEN1
CALML5	ANKRD54	MRPS14	ARMCX3	FTH1P18	ARTN	P2RX7	ARF3	MMGT1
CEND1	ANXA2R	MSANTD3	ASCC3	HECTD4	ATP5F1	PADI1	ARHGEF6	MPI
CHP1	ARHGEF10L	MTRNR2L2	ATF4	HS6ST1	ATP6V0C	PBRM1	ATP6AP2	NEK2
CLN6	ARHGEF40	MYO18A	ATP6AP1	HSPA5	AURKAIP1	PDILT	ATP6V0A1	NIPAL2
DOK3	ATG3	NIF3L1	ATP6AP2	IFT27	BAK1	PDZK1	AXL	NIT1
FXR1	ATP6V1G1	NKX1-1	ATP6V0B	ISG15	BMP8B	PHIP	BET1	NPC2
GCOM1	BAALC	NMS	ATP6V0C	JAG2	C2orf16	PMPCA	BPY2	OR4K13
LARP7	C10orf35	NRSN1	ATP6V0D1	KIAA0040	CALCOCO2	POLR3C	BPY2B	OSTC
LMO7	C11orf52	NUDT19	ATP6V1A	KRTAP19-8	CERS1	PPARGC1B	C1ORF227	PER3
LMOD3	C14orf119	NUGGC	ATP6V1B2	MIDN	CLECL1	PPP2R3C	CCDC171	PLA2G16
LYAR	C16orf70	ODF3L1	ATP6V1C1	MMGT1	CNKSR1	PRAF2	CPO	PPP2R5A
MMGT1	C19orf57	OR10AG1	ATP6V1D	MSMO1	COMMD7	PRPS2	CT47A4	PRAC1
MSI1	C1orf116	OR10T2	ATP6V1E1	NBPF9	COPB2	PSMC3	CTTNBP2NL	RAB5C
PRAF2	C21orf91	OR5AS1	ATP6V1H	NDST1	COPS2	PSMD4	CXORF22	RABEP1
RRAGD	CENPH	OST4	ATP8B4	NGB	CTSF	PTPRT	DACT2	RABGEF1
STT3A	CFAP47	OXGR1	B3GALT6	NPVF	DNAJC24	RNF115	DCTPP1	RAI14
TMEM41B	CHD9	PARP9	B4GALT7	NUDT18	DNM2	RPL23	DPM1	RFX4
XIRP2	CLDN20	PLAC8L1	C14orf169	OS9	EDC4	RPL37A	E4F1	RTKN
YIPF4	CLSTN2	PLEKHM3	C3orf58	PAPSS1	EFCAB4B	RPP21	ECM2	SAA2
ZC3HAV1	CPVL	PPAN	CA4	PHPT1	EHHADH	RPS15A	EMC1	SH3GLB2
	CSDC2	PRB2	CD302	PLAC4	ELOVL7	RSL24D1	EMC2	SIPA1L3
	CSMD3	PRRT3	CKMT2	PRAC2	EMC1	SCGB1D1	EMC3	SLC9A3
	CYB561A3	PRY2	CLK2	PTPN2	EMC6	SCYL1	EMC4	SLCO4C1
	CYP26B1	QRICH1	COG1	RNASEK	EPHB3	SDAD1	EMC6	SND1
	DCDC5	RAB42	COG2	RYBP	ESM 1	SLC25A3	EMC7	SPATA31C2
	DCP1B	RASSF3	COG3	SCARB1	FAM178A	SMPD4	EMC8	SPTBN2
	DCUN1D3	RHOU	COG4	SEL1L	FAM200B	SNRPB	EXT1	SSR2
	DERL2	RIMKLA	COG5	SLC22A20	FBXO4	SPATA16	EXTL3	SSR3
	DNAH7	RNF152	COG6	SLC28A3	FUNDC1	SPCS3	FAM179B	STARD10
	DNAJB8	SBK3	COG7	SLC35B2	FXR1	STOM	FAM43B	STMN4
	ELK3	SCAMP5	COG8	SLC39A9	GHRHR	SV2C	GAD1	STT3A
	EMC2	SIAH3	CSAG3	SOCS3	GK5	TBX2	GLP2R	TAF7L
	EMC3	SNX30	CTAG2	SPATA31C1	GNB2L1	TGFB3	GRIN3A	TGFBRAP1
	EPHA10	SRGAP2B	DCAF7	SPATA8	HEATR1	THAP2	HDLBP	THUMPD2
	FAM78A	SSR2	DDX3X	SPON1	IMPDH2	TMEM108	HEBP2	TMEM2
	FGFBP2	SSR3	DERL1	SPTLC1	IPO9	TMTC3	HHIP	TRAM1
	GABBR2	STK33	DERL2	SRPRB	IQGAP3	TRIM35	HIBCH	TRIM16
	GATA5	STRN3	DERL3	SSR1	LSM2	TRNT1	MIR-4429	TSNAX
	GCNT7	STT3A	DNAJB3	SSR2	LSM5	TROVE2	MIR-451A	TSPY2
	GJD2	SULT1C4	DNAJC10	SSR3	MATK	TSR2	MIR-451B	TSPY4
	GLTPD2	SV2A	EDDM3A	SSR4	MED6	TUBA1B	MIR-944	VCX2
	GNS	TET3	EDDM3B	STAT1	MIA	UBQLN1	HSF5	VPS45
	GPR33	THUMPD2	EDEM1	STAT2	MMGT1	WDR77	IFRD2	WDR7
	GPX6	TLR9	EDEM2	STAT3	MTA2	YWHAH	IQCB1	ZFYVE20
	GTF2F1	TMC7	EDEM3	STT3A	NKX2-8	ZNF584	ISG15	ZNF540
	IL17F	TMEM150B	EHMT2	SUDS3	NOL8	ZNF705D	KIAA1147	ZNF567
	IL27	TMEM176A	EIF2AK1	SYVN1	NPFF	ZNF845	KRTAP20-2	ZNF71
	IRGQ	TMEM41B	EIF2AK2	TM2D3	NR1H3	ZSWIM4	LRRC29	ZNF844
	ITGB5	TMPRSS11F	EIF2AK3	TM9SF2				
	KATNAL1	TNFAIP8L2	EIF2AK4	TMEM165				
	KIAA1522	TOR4A	EMC1	TMEM199				
	LACC1	TPT1	EMC10	TP53				
	LDLRAD1	TRAM1	EMC2	TXNRD3				
	LHX9	UBE2G2	EMC3	UBE2G2				
	LOXL2	UBE2J1	EMC4	UBE2J2				
	LRRC61	URAD	EMC6	UGDH				
	LY6K	USP43	EMC7	UHRF1				
	LYPD8	VIPAS39	EMC8	USP17L7				
	LYRM2	WFDC12	EMC9	UXS1				
	MAGEL2	WIPF3	ERLEC1	VMA21				
	MGAM	XYLT2	EXT1	WDR7				
	MMGT1	YDJC	EXT2	ZBED5				
	MORC2	ZNF805	EXTL3	ZNF761				

PERK-mediated UPR regulates the expression of genes involved in apoptosis, redox, amino acid transport and autophagy through eIF2 phosphorylation and the transcription factor ATF4. ZIKV infection significantly upregulates seven genes of the Reactome pathway “ATF4 activates genes in response to endoplasmic reticulum stress” ( Figure 5B), including the ERAD gene HERPUD1 and the transcription factors ATF3, CEBPB and CEBPG. GADD34 (PPP1R15A), which usually dephosphorylates eIF2α in a negative feedback loop, is significantly upregulated here by ZIKV infection, and ZIKV induces eIF2 phosphorylation in SK-N-SH cells. ^55^ ZIKV likely upregulates GADD34 to combat ER stress-induced translational repression, as fresh virions require *de novo* protein synthesis. C/EBP homologous protein (CHOP) (DDIT3) is a pro-apoptotic protein downstream of the PERK UPR pathway that others have observed to be significantly upregulated in SH-SY5Y and SK-N-SH cells in response to ZIKV infection. ^14^^,^^55^^,^^56^ CHOP induces apoptotic markers, including Caspase 3, leading to cell death. Notably, multiple PERK-mediated UPR genes (ATF4, EIF2AK1, EIF2AK2, EIF2AK3 and EIF2AK4) are ZIKV dependency factors in hiPSC-NPCs ( Table 4).

Our data mining, integration and re-analysis suggests that ZIKV specifically upregulates and is dependent on the IRE1 and PERK branches of the UPR ER stress response in SH-SY5Y cells; observations that are supported by others. ^55^^,^^56^

### ZIKV is dependent on the EMC in neuroblastoma cells

**Figure 6.**
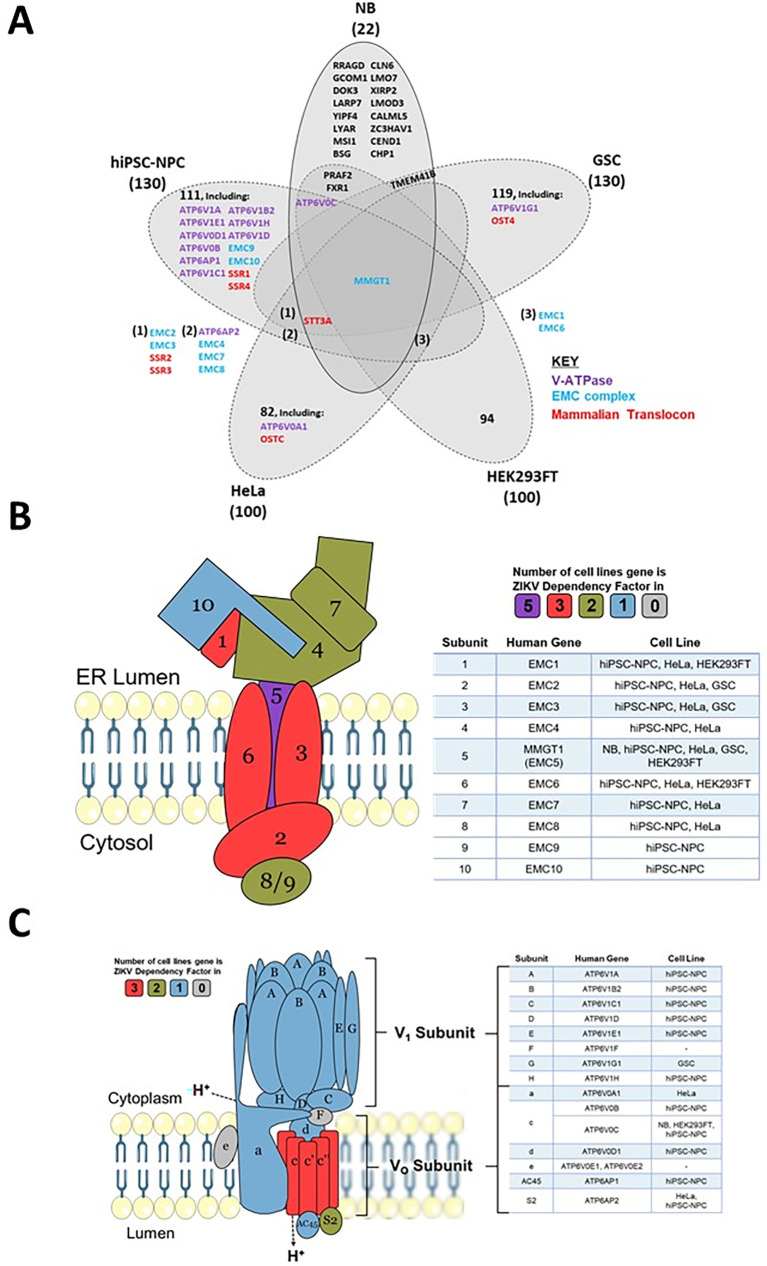
ZIKV dependency factors. Venn diagram of known ZIKV dependency factors across NB, GSC, hiPSC-NPC (NPC), HEK293FT and HeLa cells, to identify shared and cell-specific factors and protein complexes that ZIKV is dependent on for infection (A). Diagram of the EMC (B) and V-ATPase (C), based on their crystal structures. For the subunits in B and C, colours are allocated based on the number of cell types in which they act as ZIKV dependency factors (cell types stated in the adjacent tables). ZIKV, Zika virus; NB, neuroblastoma; GSC, glioma stem cell; EMC, endoplasmic reticulum membrane protein complex.

To determine which host mechanisms ZIKV may be dependent on, we cross-referenced the 22 known proteins that ZIKV requires to infect neuroblastoma cells with ZIKV dependency factors from four cell lines ( Figure 6A and Table 4). Between 72–94% of the dependency factors identified across the five different cell lines are cell-specific, highlighting how ZIKV utilises differing host factors across different cell types for its life cycle. The sparse overlap of ZIKV dependency factors identifies only one factor common to all five cell types. MMGT1 (EMC5) is a key component of the Endoplasmic Reticulum (ER) Membrane Protein Complex (EMC). The EMC is a hetero-oligomer composed of 10 subunits, has chaperone properties by assisting multi-transmembrane protein folding, and is implicated in ER stress, flavivirus infection and lipid trafficking. ^57^ To assess if ZIKV is dependent on additional EMC subunits during infection, we searched for them in our ZIKV dependency factor dataset. Our integration of varying ZIKV-dependency factor datasets show ZIKV to have a strong dependence on the EMC independent of cell type; all 10 EMC proteins are ZIKV-dependency factors in hiPSC-NPC, eight are in HeLa and three in GSC and HEK293FT cells ( Figure 6B). The EMC facilitates the expression of ZIKV transmembrane proteins (NS2B, NS4A and NS4B), ZIKV NS4B interacts with EMC subunits, and disrupting the EMC impedes infection by ZIKV and other flaviviruses. ^58^^,^^59^ We propose that the EMC stabilises ZIKV proteins through integration into the ER membrane, thus permitting efficient infection in neuroblastoma cells. If investigated, we predict that additional EMC subunits would present as ZIKV dependency factors in neuroblastoma cells.

### ZIKV is dependent on the V-ATPase in neuroblastoma cells

Acidification of the endosomal-lysosomal system by the V-ATPase is a property that viruses can utilise to drive the release of their nucleocapsid into the cytosol. ATP6V0C, a central component of the V-ATPase, is a ZIKV-dependency factor in neuroblastoma, hiPSC-NPC and HEK293FT cells ( Figure 6A). A total of 12 additional V-ATPase subunits are ZIKV dependency factors across GSC, hiPSC-NPC and HeLa cells ( Figure 6C). These 13 genes consist of multiple subunits from the Vo proton translocation and V1 ATP hydrolytic domains, identifying a functional dependence of ZIKV on the entire V-ATPase complex. The V-ATPase inhibitor Bafilomycin A1 specifically binds ATP6V0C and through V-ATPase inhibition prevents lysosomal acidification and the autophagy-lysosome pathway. ^60^ Bafilomycin A1 inhibits ZIKV infection of SH-SY5Y cells, supporting our observation ( Table 3). siRNA silencing of the V-ATPase significantly impairs ZIKV infection of T98G glioblastoma cells, collaborating its requirement for infection of nervous system tumour cells. ^61^ We propose that loss of V-ATPase function impairs ZIKV infection in SH-SY5Y cells due to a perturbed pH gradient in the endosomal system. This likely prevents fusion of the viral envelope with the endosomal membrane for release of the nucleocapsid, therefore, trapping ZIKV for degradation in the lysosome, as observed in Vero cells. ^62^

### ZIKV NS4B possesses oncolytic capability against neuroblastoma cells

**Figure 7.**
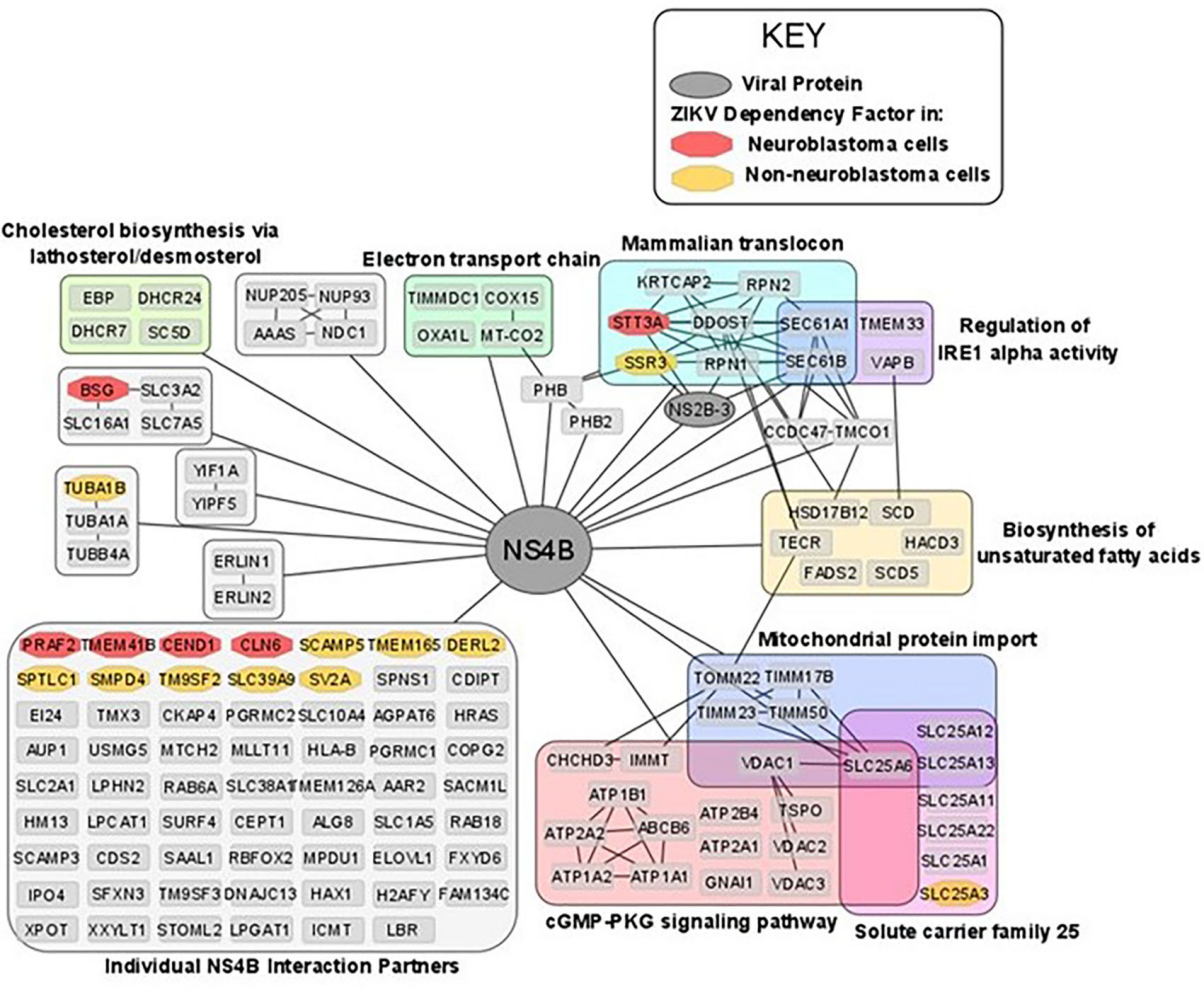
The ZIKV NS4B interactome in neuroblastoma cells and its interaction with ZIKV dependency factors. Nodes are grouped and labelled according to any sets of high-confidence interactions between the host proteins, and by pathways they are involved in. Cross-referencing the interactome with the ZIKV dependency factor datasets identifies the interaction of NS4B with dependency factors in neuroblastoma cells (red) and in GSC, hiPSC-NPC, HeLa and HEK293FT cells (collectively termed non-neuroblastoma cells, orange). To aid visualisation, any nodes possessing no high confidence interactions, other than their interaction with NS4B, have been grouped. All nodes within Figure 7 interact with NS4B, thus, to aid visualisation all edges between NS4B and nodes within a group have been condensed into a single edge between NS4B and the grouped set of nodes. ZIKV, Zika virus; NS4B, non-structural protein 4B; GSC, glioma stem cell.

ZIKV NS4B protein is principally responsible for the oncolytic effect in SH-SY5Y cells, *via* activating the mitochondrial apoptotic pathway. ^63^ Determining the interactions and mechanisms underpinning this may yield opportunities to develop a paediatric neuroblastoma therapy based on ZIKV NS4B. ZIKV NS4B has 130 known host interaction partners in SK-N-BE2 neuroblastoma cells. ^30^ Re-analysing this interactome, we observe multiple pathways previously implicated during ZIKV infection of neuroblastoma cells: including mitochondrial-, lipid metabolism- and ER-associated processes ( Figure 7). ZIKV NS4B interacts with 10 lipid biosynthesis proteins, three (SCD, SC5D and DHCR7) of which are expressed in response to SREBP pathway activation. This identifies a direct interaction between ZIKV and host lipid metabolism and the SREBP pathway, supporting our previous observations at the transcriptome level.

ZIKV NS4B recruits BAX to the mitochondria, triggers its activation, and releases Cytochrome c from mitochondria to induce mitochondrial cell death in SH-SY5Y cells. ^63^ ZIKV NS4B interacts with a multitude of mitochondrial genes ( Figure 7). Including, electron transport chain proteins (TIMMDC1, MT-CO2, COX15 and OXA1L), mitochondrial translocases that import proteins into the mitochondrial matrix (TOMM22, TIMM23, TIMM50 and TIMM17B) and Solute Carrier Family 25 members for transport of solutes across the mitochondrial membrane (SLC25A1, SLC25A3, SLC25A6, SLC25A11, SLC25A12, SLC25A13 and SLC25A22). Specifically, MT-CO2, COX15 and OXA1L are conserved catalytic core, assembly and accessory subunits of the Cytochrome c oxidase complex, respectively. The Cytochrome c oxidase complex tightly couples Cytochrome c to the inner mitochondrial membrane. We propose that NS4B interacts with Cytochrome c oxidase to uncouple it from Cytochrome c, causing Cytochrome c release through the BAX pore into the cytosol to drive the mitochondrial cell death pathway in neuroblastoma cells.

### ZIKV NS4B interacts with the Mammalian Translocon in neuroblastoma cells

ZIKV NS4B interacts with and is dependent on multiple proteins of the Mammalian Translocon ( Figures 5A and 6). The mammalian translocon is primarily composed of the Oligosaccharyl Transferase (OST) complex, the Sec61 complex and the translocon-associated protein (TRAP) complex. ^64^ The multimeric OST complex co-translationally N-glycosylates proteins within the ER to assist protein folding, stability and trafficking. The Sec61 complex, a heterotrimer of Sec61α, Sec61β and Sec61γ, co-translationally translocates newly synthesised proteins across the ER and during ER stress can regulate IRE1α activity. TRAP is a heterotetramer of SSR1, SSR2, SSR3 and SSR4 that assists co-translational translocation of proteins into the ER and can prevent aberrant N-linked glycosylation during ER stress.

STT3A, a principal component of the OST complex, interacts with ZIKV NS4B and is a ZIKV dependency factor in neuroblastoma, GSC, hiPSC-NPC and HeLa cells ( Figures 5A and 6). Regarding the additional OST subunits, ZIKV NS4B interacts with DDOST, RPN1, RPN2 and KRTCAP2, and OSTC and OST4 are ZIKV dependency factors in HeLa cells and GSCs, respectively ( Figures 5A and 6). Two forms of the OST complex exist, the STT3A and STT3B OST paralogs. KRTCAP2 and OSTC are STT3A-specific OST factors, which permit interaction of STT3A with the translocon, whilst TUSC3 and MAGT1 are STT3B-specific OST factors. ^64^ STT3A and both STT3A-specific OST factors are ZIKV interaction partners and/or dependency factors, but neither STT3B nor the STT3B-specific OST factors are. In addition to the OST factors, multiple N-linked glycosylation-related proteins (DPM1, DERL3, SYVN1, UBE2G2 and UBE2J1) are also ZIKV dependency factors in non-neuroblastoma cells ( Table 4). The OST complex inhibitor NGI-1 blocks ZIKV infection of Huh7 cells, and disrupting ZIKV prM and E protein N-glycosylation impairs the release of infectious ZIKV particles from Vero cells. ^65^^,^^66^ We propose that STT3A functions as a bonafide ZIKV dependency factor in multiple cell types, and propose that efficient infection of neuroblastoma cells by ZIKV is likely dependent on the STT3A OST paralog for N-glycosylation of its viral proteins.

ZIKV NS4B interacts with SEC61A1 and SEC61B of the Sec61 complex in neuroblastoma cells and the Sec61α inhibitor Mycolactone impedes ZIKV infection of HeLa cells. ^67^ ZIKV NS4B interacts with SSR3 of the TRAP complex in neuroblastoma cells and ZIKV is dependent on at least two of the four TRAP complex subunits for infection of GSC, hiPSC-NPC and HeLa cells ( Figure 6A). Further supporting our observation of ZIKV interacting and being dependent on the mammalian translocon is its dependence on SRPRB, SPCS3 and TRAM1; subunits of the Signal Recognition Particle (SRP), the Signal Peptidase Complex (SPCS) and the Translocating chain-associated membrane protein (TRAM), respectively. Notably, the viral protease NS2B-3 also interacts with subunits of the OST complex (STT3A, RPN1), Sec61 complex (SEC61B) and TRAP complex (SSR3) ( Figure 7). These interactions likely facilitate the co-translational cleavage of the viral polypeptide by NS2B-3 into its individual viral proteins.

From our re-analysis of a published ZIKV interactome we propose ZIKV NS4B and NS2B-3 to interact with the core complexes of the mammalian translocon, and hypothesise that ZIKV infection in neuroblastoma cells may be dependent on these interactions. The dependency of ZIKV likely stems from the mammalian translocon facilitating viral polyprotein co-translational translocation, viral polyprotein cleavage, viral membrane protein insertion and/or viral protein N-glycosylation. Additionally, ZIKV may utilise its protein interactions with the Sec61 complex, TMEM33 and VAPB, to regulate the IRE1- and PERK-mediated UPR ER stress responses, that we observed to be significantly upregulated at the transcriptome level in ZIKV infected-neuroblastoma cells.

## Conclusions

**Figure 8.**
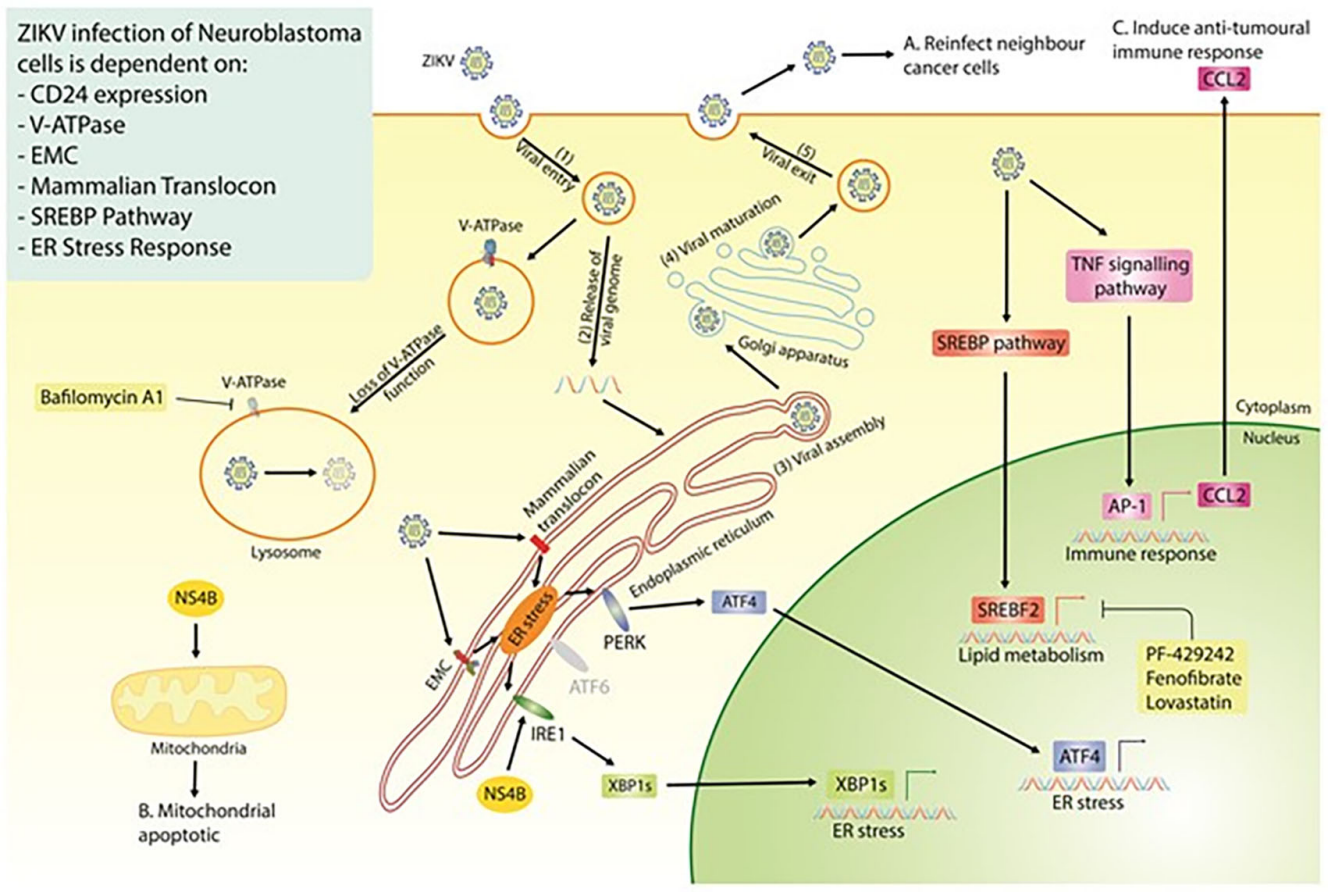
Diagram of the proposed ZIKV life cycle in neuroblastoma cells (Step 1-5), with a summary of all the currently known dependencies that the virus has for infection of neuroblastoma cells. Highlighted are the three essential properties of an oncolytic virus; the production of fresh viral particles to infect additional cancer cells (A), the ability to induce cancer cell death (B) and a mechanism through which ZIKV may induce an anti-tumoral immune response (C). ZIKV, Zika virus; EMC, endoplasmic reticulum membrane protein complex; SREBP, sterol regulatory element binding protein; ER, endoplasmic reticulum; NS4B, non-structural protein 4B.

Our study highlights the strong therapeutic potential of ZIKV, specifically the PRVABC59 strain, against multiple neuroblastoma cell-lines. Our data mining, integration and re-analysis suggest ZIKV to interact with, and be dependent on, multiple host protein complexes and pathways for its life cycle in paediatric neuroblastoma cells and for inducing oncolysis ( Figure 8). Although this area of research is still at an early stage, our extensive survey of neuroblastoma ZIKV infection studies clearly demonstrates the potential of a ZIKV-based therapeutic. There are a few avenues that need to be addressed to progress this area of research, including; (1) assessing ZIKV’s oncolytic effect against neuroblastoma in xenograft mouse models, (2) assessing ZIKV’s capability to induce an anti-tumoral immune response against neuroblastoma in immune-competent *in vivo* models, and (3) considering the effectiveness and safety of employing different forms of ZIKV-based therapeutics against neuroblastoma. Examples of the latter may include live attenuated ZIKV strains or the construction of a virotherapy that collectively expresses ZIKV NS4B and CCL2, which we observe here to hold elements of ZIKV’s oncolytic and immune activation potential, respectively.

## Data availability

### Underlying data

European Nucleotide Archive: Asian Zika virus isolate significantly changes the transcriptional profile and alternative RNA splicing events in a neuroblastoma cell line. Accession number PRJNA630088 ( https://www.ebi.ac.uk/ena/browser/view/PRJNA630088). ^80^

## References

[1] Johnsen JI Dyberg C Wickström M : Neuroblastoma—A Neural Crest Derived Embryonal Malignancy. *Front. Mol. Neurosci.* 2019;12:9. 10.3389/fnmol.2019.00009 30760980 PMC6361784

[2] Campos Cogo S Farias G Costa do Nascimento T da : An overview of neuroblastoma cell lineage phenotypes and in vitro models. *Exp. Biol. Med. (Maywood).* 2020 Dec;245(18):1637–1647. 10.1177/1535370220949237 32787463 PMC7802384

[3] Irwin MS Naranjo A Zhang FF : Revised Neuroblastoma Risk Classification System: A Report From the Children’s Oncology Group. *J. Clin. Oncol.* 2021 Jul 28 [cited 2021 Dec 13];39:3229–3241. 10.1200/JCO.21.00278 34319759 PMC8500606

[4] Chung C Boterberg T Lucas J : Neuroblastoma. *Pediatr. Blood Cancer.* 2021;68(S2):e28473. 10.1002/pbc.28473 33818884 PMC8785544

[5] Macedo N Miller DM Haq R : Clinical landscape of oncolytic virus research in 2020. *J. Immunother. Cancer.* 2020 Oct 1;8(2):e001486. 10.1136/jitc-2020-001486 33046622 PMC7552841

[6] Li C Xu D Ye Q : Zika Virus Disrupts Neural Progenitor Development and Leads to Microcephaly in Mice. *Cell Stem Cell.* 2016 Jul 7;19(1):120–126. 10.1016/j.stem.2016.04.017 27179424

[7] Tang H Hammack C Ogden SC : Zika Virus Infects Human Cortical Neural Precursors and Attenuates Their Growth. *Cell Stem Cell.* 2016 May 5;18(5):587–590. 10.1016/j.stem.2016.02.016 26952870 PMC5299540

[8] Musso D Ko AI Baud D : Zika Virus Infection — After the Pandemic. Longo DL, editor. *N. Engl. J. Med.* 2019 Oct 10;381(15):1444–1457. 10.1056/NEJMra1808246 31597021

[9] Adachi K Nielsen-Saines K : Zika Clinical Updates: Implications for Pediatrics. *Curr. Opin. Pediatr.* 2018 Feb;30(1):105–116. 10.1097/MOP.0000000000000582 29176498 PMC5798463

[10] Zhu Z Mesci P Bernatchez JA : Zika Virus Targets Glioblastoma Stem Cells through a SOX2-Integrin αvβ5 Axis. *Cell Stem Cell.* 2020 Feb;26(2):187–204.e10. 10.1016/j.stem.2019.11.016 31956038 PMC9628766

[11] Kaid C Goulart E Caires-Junior LC : Zika Virus Selectively Kills Aggressive Human Embryonal CNS Tumor Cells in vitro and In Vivo. *Cancer Res.* 2018 Jun 15;78(12):3363–3374. 10.1158/0008-5472.CAN-17-3201 29700002

[12] Kaid C Madi RA d S Astray R : Safety, Tumor Reduction, and Clinical Impact of Zika Virus Injection in Dogs with Advanced-Stage Brain Tumors. *Mol. Ther.* 2020 May;28(5):1276–1286. 10.1016/j.ymthe.2020.03.004 32220305 PMC7210722

[13] Mazar J Li Y Rosado A : Zika virus as an oncolytic treatment of human neuroblastoma cells requires CD24. *PLoS One.* 2018;13(7):e0200358. 10.1371/journal.pone.0200358 30044847 PMC6059425

[14] Bonenfant G Meng R Shotwell C : Asian Zika Virus Isolate Significantly Changes the Transcriptional Profile and Alternative RNA Splicing Events in a Neuroblastoma Cell Line. *Viruses.* 2020 May 5;12(5):E510. 10.3390/v12050510 PMC7290316 32380717

[15] Babraham Bioinformatics - FastQC A Quality Control tool for High Throughput Sequence Data:[cited 2023 Mar 29]. Reference Source

[16] LaMar D : FastQC. 2015.

[17] Kim D Paggi JM Park C : Graph-based genome alignment and genotyping with HISAT2 and HISAT-genotype. *Nat. Biotechnol.* 2019 Aug;37(8):907–915. 10.1038/s41587-019-0201-4 31375807 PMC7605509

[18] Danecek P Bonfield JK Liddle J : Twelve years of SAMtools and BCFtools. *GigaScience.* 2021 Feb 16;10(2):giab008. 10.1093/gigascience/giab008 33590861 PMC7931819

[19] Liao Y Smyth GK Shi W : featureCounts: an efficient general purpose program for assigning sequence reads to genomic features. *Bioinforma. Oxf. Engl.* 2014 Apr 1;30(7):923–930. 10.1093/bioinformatics/btt656 24227677

[20] Love MI Huber W Anders S : Moderated estimation of fold change and dispersion for RNA-seq data with DESeq2. *Genome Biol.* 2014 Dec 5;15(12):550. 10.1186/s13059-014-0550-8 25516281 PMC4302049

[21] Sherman BT Hao M Qiu J : DAVID: a web server for functional enrichment analysis and functional annotation of gene lists (2021 update). *Nucleic Acids Res.* 2022 Mar 23;50(W1):W216–W221. 10.1093/nar/gkac194 35325185 PMC9252805

[22] Huang DW Sherman BT Lempicki RA : Systematic and integrative analysis of large gene lists using DAVID bioinformatics resources. *Nat. Protoc.* 2009;4(1):44–57. 10.1038/nprot.2008.211 19131956

[23] Gene Ontology Consortium: The Gene Ontology resource: enriching a GOld mine. *Nucleic Acids Res.* 2021 Jan 8;49(D1):D325–D334. 10.1093/nar/gkaa1113 33290552 PMC7779012

[24] Ashburner M Ball CA Blake JA : Gene ontology: tool for the unification of biology. The Gene Ontology Consortium. *Nat. Genet.* 2000 May;25(1):25–29. 10.1038/75556 10802651 PMC3037419

[25] Kanehisa M Goto S : KEGG: kyoto encyclopedia of genes and genomes. *Nucleic Acids Res.* 2000 Jan 1;28(1):27–30. 10.1093/nar/28.1.27 10592173 PMC102409

[26] Kanehisa M Furumichi M Sato Y : KEGG for taxonomy-based analysis of pathways and genomes. *Nucleic Acids Res.* 2023 Jan 6;51(D1):D587–D592. 10.1093/nar/gkac963 36300620 PMC9825424

[27] Kanehisa M : Toward understanding the origin and evolution of cellular organisms. *Protein Sci. Publ. Protein Soc.* 2019 Nov;28(11):1947–1951. 10.1002/pro.3715 31441146 PMC6798127

[28] Luo W Pant G Bhavnasi YK : Pathview Web: user friendly pathway visualization and data integration. *Nucleic Acids Res.* 2017 Jul 3;45(W1):W501–W508. 10.1093/nar/gkx372 28482075 PMC5570256

[29] Orchard S Ammari M Aranda B : The MIntAct project—IntAct as a common curation platform for 11 molecular interaction databases. *Nucleic Acids Res.* 2014 Jan 1;42(Database issue):D358–D363. 10.1093/nar/gkt1115 24234451 PMC3965093

[30] Scaturro P Stukalov A Haas DA : An orthogonal proteomic survey uncovers novel Zika virus host factors. *Nature.* 2018 Sep;561(7722):253–257. 10.1038/s41586-018-0484-5 30177828

[31] Szklarczyk D Gable AL Lyon D : STRING v11: protein-protein association networks with increased coverage, supporting functional discovery in genome-wide experimental datasets. *Nucleic Acids Res.* 2019 Jan 8;47(D1):D607–D613. 10.1093/nar/gky1131 30476243 PMC6323986

[32] Shannon P Markiel A Ozier O : Cytoscape: a software environment for integrated models of biomolecular interaction networks. *Genome Res.* 2003 Nov;13(11):2498–2504. 10.1101/gr.1239303 14597658 PMC403769

[33] Wang S Zhang Q Tiwari SK : Integrin αvβ5 Internalizes Zika Virus during Neural Stem Cells Infection and Provides a Promising Target for Antiviral Therapy. *Cell Rep.* 2020 Jan;30(4):969–983.e4. 10.1016/j.celrep.2019.11.020 31956073 PMC7293422

[34] Li Y Muffat J Javed AO : Genome-wide CRISPR screen for Zika virus resistance in human neural cells. *Proc. Natl. Acad. Sci.* 2019 May 7;116(19):9527–9532. 10.1073/pnas.1900867116 31019072 PMC6510995

[35] Savidis G McDougall WM Meraner P : Identification of Zika Virus and Dengue Virus Dependency Factors using Functional Genomics. *Cell Rep.* 2016 Jun 28;16(1):232–246. 10.1016/j.celrep.2016.06.028 27342126

[36] Pereira R Costa V Gomes G : Anti-Zika virus activity of plant extracts containing polyphenols and triterpenes on Vero CCL-81 and human neuroblastoma SH-SY5Y cells. *Chem. Biodivers.* 2022 Mar 13. 10.1002/cbdv.202100842 35285139

[37] Kedarinath K Fox CR Crowgey E : CD24 Expression Dampens the Basal Antiviral State in Human Neuroblastoma Cells and Enhances Permissivity to Zika Virus Infection. *Viruses.* 2022 Aug 6;14(8):1735. 10.3390/v14081735 36016357 PMC9416398

[38] Anfasa F Siegers JY Kroeg M van der : Phenotypic Differences between Asian and African Lineage Zika Viruses in Human Neural Progenitor Cells. *mSphere.* 2017 Aug;2(4). 10.1128/mSphere.00292-17 28815211 PMC5555676

[39] Jorgačevski J Korva M Potokar M : ZIKV Strains Differentially Affect Survival of Human Fetal Astrocytes versus Neurons and Traffic of ZIKV-Laden Endocytotic Compartments. *Sci. Rep.* 2019 May 30 [cited 2020 May 2];9:8069. 10.1038/s41598-019-44559-8 31147629 PMC6542792

[40] Hu H Zhang W Huang D : Clinical characteristics, treatment and prognosis of paediatric patients with metastatic neuroblastoma to the brain. *Clin. Neurol. Neurosurg.* 2019 Sep 1;184:105372. 10.1016/j.clineuro.2019.105372 31155296

[41] Ramond A Lobkowicz L Clemente NS : Postnatal symptomatic Zika virus infections in children and adolescents: A systematic review. PLoS Negl. Trop. Dis. 2020 Oct 2;14(10): e0008612. 10.1371/journal.pntd.0008612 33006989 PMC7556487

[42] Wen F Armstrong N Hou W : Zika virus increases mind bomb 1 levels, causing degradation of pericentriolar material 1 (PCM1) and dispersion of PCM1-containing granules from the centrosome. *J. Biol. Chem.* 2019 Dec 6;294(49):18742–18755. 10.1074/jbc.RA119.010973 31666336 PMC6901299

[43] Gazon H Barbeau B Mesnard JM : Hijacking of the AP-1 Signaling Pathway during Development of ATL. *Front. Microbiol.* 2018 [cited 2022 Mar 29];8. 10.3389/fmicb.2017.02686 29379481 PMC5775265

[44] Atsaves V Leventaki V Rassidakis GZ : AP-1 Transcription Factors as Regulators of Immune Responses in Cancer. *Cancers.* 2019 Jul 23;11(7):E1037. 10.3390/cancers11071037 PMC6678392 31340499

[45] Lima MC Mendonça LR de Rezende AM : The Transcriptional and Protein Profile From Human Infected Neuroprogenitor Cells Is Strongly Correlated to Zika Virus Microcephaly Cytokines Phenotype Evidencing a Persistent Inflammation in the CNS. *Front. Immunol.* 2019 [cited 2020 Jul 9];10. 10.3389/fimmu.2019.01928/full 31474994 PMC6707094

[46] Braz-De-Melo HA Pasquarelli-do-Nascimento G Corrêa R : Potential neuroprotective and anti-inflammatory effects provided by omega-3 (DHA) against Zika virus infection in human SH-SY5Y cells. *Sci. Rep.* 2019 Dec 27 [cited 2020 May 2];9:20119. 10.1038/s41598-019-56556-y 31882804 PMC6984748

[47] Parker JN Meleth S Hughes KB : Enhanced inhibition of syngeneic murine tumors by combinatorial therapy with genetically engineered HSV-1 expressing CCL2 and IL-12. *Cancer Gene Ther.* 2005 Apr;12(4):359–368. 10.1038/sj.cgt.7700784 15678154

[48] Osuna-Ramos JF Reyes-Ruiz JM Ángel RM del : The Role of Host Cholesterol During Flavivirus Infection. *Front. Cell. Infect. Microbiol.* 2018 Nov 2;8:388. 10.3389/fcimb.2018.00388 30450339 PMC6224431

[49] Raini SK Takamatsu Y Dumre SP : The novel therapeutic target and inhibitory effects of PF-429242 against Zika virus infection. *Antivir. Res.* 2021 Aug;192:105121. 10.1016/j.antiviral.2021.105121 34175321

[50] Weber LW Boll M Stampfl A : Maintaining cholesterol homeostasis: Sterol regulatory element-binding proteins. *World J. Gastroenterol: WJG.* 2004 Nov 1;10(21):3081–3087. 10.3748/wjg.v10.i21.3081 15457548 PMC4611246

[51] Sabino C Basic M Bender D : Bafilomycin A1 and U18666A Efficiently Impair ZIKV Infection. *Viruses.* 2019 Jun 6;11(6). 10.3390/v11060524 31174294 PMC6630673

[52] Mlera L Offerdahl DK Dorward DW : The liver X receptor agonist LXR 623 restricts flavivirus replication. *Emerg. Microbes Infect.* 2021 Jun 24;10:1378–1389. 10.1080/22221751.2021.1947749 34162308 PMC8259867

[53] Grefhorst A Elzinga BM Voshol PJ : Stimulation of lipogenesis by pharmacological activation of the liver X receptor leads to production of large, triglyceride-rich very low density lipoprotein particles. J. Biol. Chem. 2002Sep 13;277(37):34182–34190. 10.1074/jbc.M204887200 12097330

[54] Gladwyn-Ng I Cordón-Barris L Alfano C : Stress-induced unfolded protein response contributes to Zika virus–associated microcephaly. *Nat. Neurosci.* 2018 Jan;21(1):63–71. 10.1038/s41593-017-0038-4 29230053

[55] Tan Z Zhang W Sun J : ZIKV infection activates the IRE1-XBP1 and ATF6 pathways of unfolded protein response in neural cells. *J. Neuroinflammation.* 2018 Sep 21 [cited 2020 Apr 30];15:275. 10.1186/s12974-018-1311-5 30241539 PMC6151056

[56] Carr M Gonzalez G Martinelli A : Upregulated expression of the antioxidant sestrin 2 identified by transcriptomic analysis of Japanese encephalitis virus-infected SH-SY5Y neuroblastoma cells. *Virus Genes.* 2019 Oct 1;55(5):630–642. 10.1007/s11262-019-01683-x 31292858

[57] Ngo AM Shurtleff MJ Popova KD : The ER membrane protein complex is required to ensure correct topology and stable expression of flavivirus polyproteins. *elife.* 8:e48469. 10.7554/eLife.48469 31516121 PMC6756788

[58] Barrows NJ Anglero-Rodriguez Y Kim B : Dual roles for the ER membrane protein complex in flavivirus infection: viral entry and protein biogenesis. *Sci. Rep.* 2019 Jul 4;9(1):9711. 10.1038/s41598-019-45910-9 31273220 PMC6609633

[59] Lin DL Inoue T Chen YJ : The ER Membrane Protein Complex Promotes Biogenesis of Dengue and Zika Virus Non-structural Multi-pass Transmembrane Proteins to Support Infection. *Cell Rep.* 2019 May;27(6):1666–1674.e4. 10.1016/j.celrep.2019.04.051 31067454 PMC6521869

[60] Mangieri LR Mader BJ Thomas CE : ATP6V0C Knockdown in Neuroblastoma Cells Alters Autophagy-Lysosome Pathway Function and Metabolism of Proteins that Accumulate in Neurodegenerative Disease. *PLoS One.* 2014 Apr 2;9(4):e93257. 10.1371/journal.pone.0093257 24695574 PMC3973706

[61] Li M Zhang D Li C : Characterization of Zika Virus Endocytic Pathways in Human Glioblastoma Cells. *Front. Microbiol.* 2020 Mar 6 [cited 2020 Jun 4];11. 10.3389/fmicb.2020.00242 Reference Source PMC7069030 32210929

[62] Owczarek K Chykunova Y Jassoy C : Zika virus: mapping and reprogramming the entry. *Cell Commun. Signal.* 2019 May 3;17(1):41. 10.1186/s12964-019-0349-z 31053158 PMC6500006

[63] Han X Wang J Yang Y : Zika Virus Infection Induced Apoptosis by Modulating the Recruitment and Activation of Proapoptotic Protein Bax. *J. Virol.* 2021 Mar 25 [cited 2021 Apr 7];95(8). 10.1128/JVI.01445-20 Reference Source 33536166 PMC8103684

[64] Braunger K Pfeffer S Shrimal S : Structural basis for coupling of protein transport and N-glycosylation at the mammalian endoplasmic reticulum. *Science.* 2018 Apr 13;360(6385):215–219. 10.1126/science.aar7899 29519914 PMC6319373

[65] Puschnik AS Marceau CD Ooi YS : A small molecule oligosaccharyltransferase inhibitor with pan-flaviviral activity. *Cell Rep.* 2017 Dec 12;21(11):3032–3039. 10.1016/j.celrep.2017.11.054 29241533 PMC5734657

[66] Gwon YD Zusinaite E Merits A : N-glycosylation in the Pre-Membrane Protein Is Essential for the Zika Virus Life Cycle. *Viruses.* 2020 Aug 23;12(9):E925. 10.3390/v12090925 PMC7552079 32842538

[67] Monel B Compton AA Bruel T : Zika virus induces massive cytoplasmic vacuolization and paraptosis-like death in infected cells. *EMBO J.* 2017 Jun 14;36(12):1653–1668. 10.15252/embj.201695597 28473450 PMC5470047

[68] Mendonça-Vieira LR de Aníbal-Silva CE Menezes-Neto A : Reactive Oxygen Species (ROS) Are Not a Key Determinant for Zika Virus-Induced Apoptosis in SH-SY5Y Neuroblastoma Cells. *Viruses.* 2021 Oct 20;13(11):2111. 10.3390/v13112111 34834918 PMC8622630

[69] Hughes BW Addanki KC Sriskanda AN : Infectivity of Immature Neurons to Zika Virus: A Link to Congenital Zika Syndrome. *EBioMedicine.* 2016 Jun 23;10:65–70. 10.1016/j.ebiom.2016.06.026 27364784 PMC5006602

[70] Bagasra O Shamabadi NS Pandey P : Differential expression of miRNAs in a human developing neuronal cell line chronically infected with Zika virus. *Libyan J. Med.* 2021 Jan 1;16(1):1909902. 10.1080/19932820.2021.1909902 33849406 PMC8049460

[71] Mlera L Bloom ME : Differential Zika Virus Infection of Testicular Cell Lines. *Viruses.* 2019 Jan 9 [cited 2020 Apr 30];11(1). 10.3390/v11010042 30634400 PMC6356326

[72] Hou W Armstrong N Obwolo LA : Determination of the Cell Permissiveness Spectrum, Mode of RNA Replication, and RNA-Protein Interaction of Zika Virus. *BMC Infect. Dis.* 2017 Mar 31 [cited 2020 May 1];17:239. 10.1186/s12879-017-2338-4 28359304 PMC5374689

[73] Castro FL Geddes VEV Monteiro FLL : MicroRNAs 145 and 148a Are Upregulated During Congenital Zika Virus Infection. *ASN Neuro.* 2019 Jan;11:175909141985098. 10.1177/1759091419850983 31213064 PMC6585135

[74] Bos S Viranaicken W Turpin J : The structural proteins of epidemic and historical strains of Zika virus differ in their ability to initiate viral infection in human host cells. *Virology.* 2018 Mar 1;516:265–273. 10.1016/j.virol.2017.12.003 29395111

[75] Sánchez-San Martín C Li T Bouquet J : Differentiation enhances Zika virus infection of neuronal brain cells. *Sci. Rep.* 2018 Sep 28 [cited 2020 Apr 30];8:14543. 10.1038/s41598-018-32400-7 30266962 PMC6162312

[76] Giel-Moloney M Goncalvez AP Catalan J : Chimeric yellow fever 17D-Zika virus (ChimeriVax-Zika) as a live-attenuated Zika virus vaccine. *Sci. Rep.* 2018 Sep 4 [cited 2020 Apr 30];8:13206. 10.1038/s41598-018-31375-9 30181550 PMC6123396

[77] Himmelsbach K Hildt E : Identification of various cell culture models for the study of Zika virus. *World J. Virol.* 2018 Feb 12;7(1):10–20. 10.5501/wjv.v7.i1.10 29468137 PMC5807893

[78] Alpuche-Lazcano SP McCullogh CR Del Corpo O : Higher Cytopathic Effects of a Zika Virus Brazilian Isolate from Bahia Compared to a Canadian-Imported Thai Strain. *Viruses.* 2018 Jan 27 [cited 2020 Apr 30];10(2). 10.3390/v10020053 29382068 PMC5850360

[79] Offerdahl DK Dorward DW Hansen BT : Cytoarchitecture of Zika virus infection in human neuroblastoma and Aedes albopictus cell lines. *Virology.* 2017 Jan;501:54–62. 10.1016/j.virol.2016.11.002 27863275 PMC5201448

[80] University At Albany: Asian Zika virus isolate significantly changes the transcriptional profile and alternative RNA splicing events in a neuroblastoma cell line.[Dataset]. *European Nucleotide Archive.* 2020. Reference Source 10.3390/v12050510 PMC7290316 32380717

