# Supplementary material for: Integrated re-analysis of transcriptomic and proteomic datasets reveals potential mechanisms for Zika viral-based oncolytic therapy in neuroblastoma
Source: F1000Res. 2024 May 21;12:719. Originally published 2023 Jun 21. [Version 3] doi: 10.12688/f1000research.132627.3 (PMC11187533; doi:10.12688/f1000research.132627.3)
Supplement: Peer review history

# Reviewer report: Reviewer response for version 3

**DOI:** 10.5256/f1000research.166562.r286184

**Published:** 2024-06-19

**Authors:** Monica Martinez Pacheco

**Affiliations:** 1 Autonomous University of Queretaro, Queretaro, Mexico

The manuscript "Integrated re-analysis of transcriptomic and proteomic datasets reveals potential mechanisms for Zika viral-based oncolytic therapy in neuroblastoma" presented by Sherwood and collaborators approaches to an exciting proposal for new and necessary alternatives to the current oncotherapy for neuroblastoma based on extensive bioinformatics analyses. Specifically, they suggest ZIKV NS4B as a likely mediator of ZIKV interaction with IRE1-mediated UPR, lipid metabolism, and mammalian translocon in neuroblastoma SH-SY5Y cells.

 Although this finding may contribute to this research field, authors should consider significant revisions before indexing. Next, I expose my comments for the author's and editors' consideration:

 1. In the Keypoints section, what do the authors mean by "Most pediatric neuroblastoma cell lines are susceptible to Zika viral infection" when they only analyzed data from two neuroblastoma cell lines? The statement should not be included as a Keypoint if it refers to previous literature on the potential Zika viral infection of neuroblastoma cell lines. Keypoints should be made based on their research results only.

 2. The authors have chosen to work with the SK-N-BE(2) cell line, a neuroblastoma cell line established from a bone marrow biopsy taken from a patient with disseminated neuroblastoma after repeated courses of chemotherapy and radiotherapy. They propose a potential ZIKV-based therapeutic for neuroblastoma. The decision to use this cell line rather than one from a non-treated patient may only partially fulfill their research strategy outcome. Analyses should be made on non-treated neuroblastoma data to understand the effect of Zika viral infection as an oncotherapy alternative.

 3. Authors must justify the usage of four different databases for enrichment analyses.

 4. Authors must include a link to the pipeline and scripts used for bioinformatic analyses. This will significantly enhance the transparency and reproducibility of their research.

 5. Literature mining results presented in the Results section could be included as a Keypoint if the authors incorporate the related methods in the Methods section.

Is the work clearly and accurately presented and does it cite the current literature?

Yes

If applicable, is the statistical analysis and its interpretation appropriate?

Yes

Are all the source data underlying the results available to ensure full reproducibility?

Partly

Is the study design appropriate and is the work technically sound?

Yes

Are the conclusions drawn adequately supported by the results?

Yes

Are sufficient details of methods and analysis provided to allow replication by others?

Partly

Reviewer Expertise:

Genomics, transcriptomics, molecular biology, genetics, cancer and complex diseases.

I confirm that I have read this submission and believe that I have an appropriate level of expertise to confirm that it is of an acceptable scientific standard, however I have significant reservations, as outlined above.

# Reviewer report: Reviewer response for version 3

**DOI:** 10.5256/f1000research.166562.r280953

**Published:** 2024-05-24

**Authors:** Thomas C. N. Leung

**Affiliations:** 1 The Chinese University of Hong Kong,, Hong Kong, China

Most of the concerns raised have been adequately addressed. However, I noticed that my comment #4 from the previous review has not been addressed, and I did not receive a response from the authors regarding this issue.

 To clarify my concern from comment #4:

 In the methods section, specifically the “ZIKV dependency factor source and analysis” subsection, the authors state, Extensive data mining revealed there are currently only 22 known ZIKV dependency factors in neuroblastoma cells, identified via a shRNA screen in paediatric SK-N-BE2 neuroblastoma cells.” This suggests that the first column of Table 4 should reflect the results from this experiment. However, the header of the first column in Table 4 indicates the use of 'SH-SY5Y' cells instead of the ‘SK-N-BE2’ cells mentioned in the methodology section.

 Clarification is needed to determine whether the discrepancy lies in the table or the methods description. Most of the concerns raised have been adequately addressed. However, I noticed that my comment #4 from the previous review has not been addressed, and I did not receive a response from the authors regarding this issue.

 To clarify my concern from comment #4:

 In the methods section, specifically the “ ***ZIKV dependency factor source and analysis***” subsection, the authors state, " ***Extensive data mining revealed there are currently only 22 known ZIKV dependency factors in neuroblastoma cells, identified via a shRNA screen in paediatric SK-N-BE2 neuroblastoma cells.***” This suggests that the first column of Table 4 should reflect the results from this experiment. However, the header of the first column in Table 4 indicates the use of ' **SH-SY5Y**' cells instead of the ‘ **SK-N-BE2**’ cells mentioned in the methodology section.

 Clarification is needed to determine whether the discrepancy lies in the table or the methods description.

Is the work clearly and accurately presented and does it cite the current literature?

Partly

If applicable, is the statistical analysis and its interpretation appropriate?

I cannot comment. A qualified statistician is required.

Are all the source data underlying the results available to ensure full reproducibility?

Yes

Is the study design appropriate and is the work technically sound?

Yes

Are the conclusions drawn adequately supported by the results?

Yes

Are sufficient details of methods and analysis provided to allow replication by others?

Partly

Reviewer Expertise:

Proteomics, Cellular Biology

I confirm that I have read this submission and believe that I have an appropriate level of expertise to confirm that it is of an acceptable scientific standard.

# Reviewer report: Reviewer response for version 2

**DOI:** 10.5256/f1000research.158746.r267294

**Published:** 2024-05-06

**Authors:** Thomas C. N. Leung

**Affiliations:** 1 The Chinese University of Hong Kong,, Hong Kong, China

The manuscript examines the potential of Zika virus (ZIKV) as an oncolytic virotherapy for neuroblastoma. Neuroblastoma is an aggressive childhood cancer and the leading cause of cancer deaths in infants. Current treatments have significant toxicity. Oncolytic virotherapy could help develop safer therapies. ZIKV interests researchers due to its ability to access the nervous system and kill neural cells.

 The study analyzes the papers examining ZIKV infection in neuroblastoma cell lines. Most cell lines are highly susceptible to ZIKV and viability reduces to 20% or less. Susceptibility is independent of clinical factors. The PRVABC59 strain shows most promise due to better RNA replication and ability to cross the blood-brain barrier. RNA sequencing of infected cells shows changes in pathways like TNF signaling, lipid metabolism and endoplasmic reticulum stress response. ZIKV increases TNF transcription factors and CCL2, pointing to an immune response.

 Analysis of interactomes identifies ZIKV NS4B interacting with 130 host proteins. Networks link NS4B to IRE1 stress response, lipid metabolism proteins and the translocon complex. The paper reviews host dependency factors identified in screens and proposes relationships to protein complexes. Modulating lipids and associated pathways alters ZIKV infection. Inhibitors of SREBP and treatments affecting late endosomes reduce infection.

 Overall, the work evaluated ZIKV’s potential as an oncolytic virotherapy for neuroblastoma through direct cytotoxicity and immunogenicity. Integrative omics uncover molecular insights into host factors and pathways regulated during infection that could inform therapeutic development. However, some clarification is needed regarding:

1. Regarding the methodology section on 'ZIKV interactome source and analysis', some additional details could aid reproducibility. The authors mention raw data was processed using MaxQuant and Andromeda, but it is unclear if they re-analyzed the raw data or obtained processed results. Providing search parameters, such as post-translational modifications considered and database details, as well as the false discovery rate threshold used, would strengthen the method description.
2. Additionally, when describing DAVID analysis of ZIKV NS4B interacting partners, more details on the specific functional analysis performed would be informative, as DAVID is commonly used for annotation rather than direct interaction identification.  
3. A few minor points regarding data presentation could also be clarified. The bar charts in Figure 1B and 1C contain numbers at the end of bars but the meaning of these numbers is unspecified. Figures 4 and 5 employ asterisks but it is unclear if they denote statistical significance levels or something else, as some bars contain double lines of asterisks.
4. Lastly, first column of Table 4 header of 'SH-SY5Y' appears inconsistent with the methods section stating analyses used the SK-N-BE2 cell line. Clarification is needed on whether the table or method description requires correction. Addressing these points of clarity would help fully evaluate the rigour and reproducibility of the work. With revision, this study could provide valuable insights into ZIKV oncolysis and neuroblastoma biology.

Is the work clearly and accurately presented and does it cite the current literature?

Partly

If applicable, is the statistical analysis and its interpretation appropriate?

I cannot comment. A qualified statistician is required.

Are all the source data underlying the results available to ensure full reproducibility?

Yes

Is the study design appropriate and is the work technically sound?

Yes

Are the conclusions drawn adequately supported by the results?

Yes

Are sufficient details of methods and analysis provided to allow replication by others?

Partly

Reviewer Expertise:

Proteomics, Cellular Biology

I confirm that I have read this submission and believe that I have an appropriate level of expertise to confirm that it is of an acceptable scientific standard, however I have significant reservations, as outlined above.

# Reviewer report: Reviewer response for version 1

**DOI:** 10.5256/f1000research.145561.r196248

**Published:** 2023-10-12

**Authors:** Estanislao Nistal-Villan, Vicent Tur-Planells

**Affiliations:** 1 Instituto de Medicina Molecular Aplicada (IMMA) Nemesio Díez, Facultad de Medicina, Universidad San Pablo-CEU, Boadilla del Monte, Spain; 2 Universidad CEU San Pablo, Madrid, Community of Madrid, Spain

**Integrative transcriptomic and proteomic study of Zika viral infection reveals potential mechanisms for oncolytic therapy in neuroblastoma.**

 The article presents an integration of transcriptomics and proteomics of several previous studies where is used the Zika virus (ZIKV) in neuroblastoma cells as a potential therapy in high-risk neuroblastoma. This pediatric cancer, characterized by its aggressive nature and limited treatment options in state-of-the-art non-responder patients, presents a pressing challenge in pediatric oncology. The authors focus on the explanation of the unique properties of ZIKV, its tropism in neuroblastoma cancer cells, and an extensive review of the host immune response in a large-scale analysis referring to previous studies.

 The study focuses on different aspects regarding ZIKV-induced cell response, trying to integrate published data to propose possible targets that are characteristic of ZIKV in neuroblastoma. However, all the hypotheses and conclusions made in the study need to be validated in normalized conditions without the bias of putting together many different studies performed in different conditions. Moreover, the article should be rewritten excluding the expressions where the authors attribute the actual empirical work of the assays. They should also state and point out that the work is collecting data from previous studies and better describe the integration methodology putting all together to offer an easier view of unequivocal unique conclusions beyond previous studies.

 Overall, the information provided in this article would be enlightening for having a general descriptive view of the different relevant stages in ZIKV infection that can elucidate an integrative understanding of strategies to improve the virotherapy but lacks scientific value since no validation of the the hypothesis is performed.

 MAJOR POINTS

- Authors should experimentally validate their bioinformatic-based hypothesis.

MINOR POINTS

- At the end of the introduction, the sentence: “Pediatric neuroblastoma, like pediatric brain tumors, are predominantly tumors of the nervous system consisting of cancerous cells with neural characteristics.” Is redundant and not necessary.
- Authors should better describe the original data used for bioinformatic analysis. Papers that study the topic are presented, but the source of the data should be better described.
- “The non-sympathetic nervous system and non-pediatric origin of the T-268 and JFEN cells likely explain their resistance to ZIKV infection, as ZIKV has a tropism for pediatric nervous system cancer cells” needs citation.
- “The neurotropism of the Asian lineage makes it the clear choice, over the African lineage, for developing ZIKV oncolytic virotherapy against brain tumors” needs citation.
- In Table 1 and Table 2, it is necessary to say in which *in vitro* viability test is based on the assay (metabolic, death markers, etc.)
-  In Table 2, the degree of infection, viral titer, and cell viability, is it normalized with the same MOI, and cancer cell type? Which cancer cell type? Would be better to explain how the authors establish the Data accordance value.
- “The multiple ZIKV pandemics identified that infection by an Asian strain is generally well accommodated by children, thus providing evidence for the safety of employing an Asian strain” needs citation.
- In Figure 3A, why authors propose that SREBF2 transcriptional pathway is responsible for the upregulation of cholesterol biosynthesis when there are other pathways with more statistical difference and fold-change.
- In the sentence “ZIKV likely remodels the cellular lipid composition to help produce a favorable environment for efficient replication”, it should be taken into account that ZIKV cannot be the one producing that, but the host viral response be responsible for those effects, and that has not been proved. It could be changed by “ZIKV infection induces all those changes…”

Is the work clearly and accurately presented and does it cite the current literature?

Partly

If applicable, is the statistical analysis and its interpretation appropriate?

I cannot comment. A qualified statistician is required.

Are all the source data underlying the results available to ensure full reproducibility?

No

Is the study design appropriate and is the work technically sound?

Partly

Are the conclusions drawn adequately supported by the results?

No

Are sufficient details of methods and analysis provided to allow replication by others?

No

Reviewer Expertise:

Innate immunity and oncolytic virus research.

We confirm that we have read this submission and believe that we have an appropriate level of expertise to state that we do not consider it to be of an acceptable scientific standard, for reasons outlined above.

# Response

**Published:** 2023-11-08

**Authors:** Rob Ewing

**Affiliations:** School of Biological Sciences, University of Southampton, Southampton, England, UK

**We thank the reviewer for the constructive critique of the manuscript. The intention of our study is to integrate and repurpose the multiple omics datasets that have been published in the literature investigating how Zika infects mammalian cells. Many of these studies have used neuroblastoma cells as a model, and we therefore see this as an opportunity to study the potential oncolytic activity of the virus in neuroblastoma, which the original aforementioned studies did not. The reviewer points out that the analysis will be strengthened by validating the hypotheses with further lab-based studies – and we agree with this. However, our intention in this paper is to layout potential molecular mechanisms that are important in the oncolytic response that can be investigated by us and others going forward. We see our paper as an important first step in establishing potential mechanisms using data mining techniques that should stand alone in a field in which there are as yet relatively few studies.** 

 MAJOR POINTS 

 • Authors should experimentally validate their bioinformatic-based hypothesis.  

- **The intent of the paper was to use in silico methods to identify molecular mechanisms involved in ZIKV infection of neuroblastoma cells. For many of the components that are highlighted by our integration and re-analysis of published datasets we have sourced and referenced functional experiments that support any propositions/conclusions which we make. Two representative examples of these include: **

- **We re-analyse RNA-Seq data of ZIKV-infected neuroblastoma cells and propose the SREBP pathway to be upregulated at the transcriptome level. We link this to data that regulation of this pathway through three different inhibitors hinders ZIKV infection in neuroblastoma cells, thus supporting the involvement of this pathway during infection.  **
- **We use ZIKV dependency factor screens to propose that ZIKV infection is dependent on the ATPase for infection in neuroblastoma cells. We back this hypothesis up with data that shows the ATPase inhibitor Bafilomycin A1 inhibits ZIKV infection in neuroblastoma **

MINOR POINTS 

 • At the end of the introduction, the sentence: “Pediatric neuroblastoma, like pediatric brain tumors, are predominantly tumors of the nervous system consisting of cancerous cells with neural characteristics.” Is redundant and not necessary. 

- **This sentence has been removed** 

 • Authors should better describe the original data used for bioinformatic analysis. Papers that study the topic are presented, but the source of the data should be better described. 

- **Additional details have been added throughout the methods section to better describe the original data. A representative example is “Scaturro et al., produced this interactome through stable expression of HA-tagged ZIKV proteins in SK-N-BE2 cells, isolation of ZIKV-host protein complexes through HA-affinity purifications, then sample preparation and run for LC–MS/MS (n = 4). 31 Raw data was processed using MaxQuant with Andromeda search engine. High-confidence interactions were determined by Bayesian statistical modelling, with log2(fold change) ≥ 2.5; unadjusted one-sided P ≤ 0.05.”** 

• “The non-sympathetic nervous system and non-pediatric origin of the T-268 and JFEN cells likely explain their resistance to ZIKV infection, as ZIKV has a tropism for pediatric nervous system cancer cells” needs citation. 

- **Citation added **

• “The neurotropism of the Asian lineage makes it the clear choice, over the African lineage, for developing ZIKV oncolytic virotherapy against brain tumors” needs citation. 

-  
**Text has been removed **

• In Table 1 and Table 2, it is necessary to say in which in vitro viability test is based on the assay (metabolic, death markers, etc.) 

- **The details of the assays have now been added to both tables **

• In Table 2, the degree of infection, viral titer, and cell viability, is it normalized with the same MOI, and cancer cell type? Which cancer cell type? Would be better to explain how the authors establish the Data accordance value.  

- **No the degree of infection, viral titer, and cell viability are not normalised by MOI or cell type. We have added columns to specify the MOI range and cell lines used. With different studies from different laboratories, it would be difficult to normalize the data by these features. Hence we prefer to present the experimental parameters as provided in the papers themselves which we used as a guide to interpretation. We also added this text to explain the ‘Data accordance’ value: **

- **“Data accordance is a qualitative measure which we employed to describedenotes the degree of similarity of the results between publications that performed ZIKV cell viability of cell death assaysinfection assays in of neuroblastoma cells using the same ZIKV strain. Data accordance of five denotes that the findings of one publication closely support the findings from another, a data accordance of one denotes publications reporting vastly contrasting results. When a viral strain is published in only one paper, it is allocated a data accordance of NA" **

“The multiple ZIKV pandemics identified that infection by an Asian strain is generally well accommodated by children, thus providing evidence for the safety of employing an Asian strain” needs citation. 

- **Citation added **

• In Figure 3A, why authors propose that SREBF2 transcriptional pathway is responsible for the upregulation of cholesterol biosynthesis when there are other pathways with more statistical difference and fold-change. 

- **Text has been added to the manuscript to help clarify why we believe the SREBF2 transcriptional pathway may contribute to the upregulation of cholesterol biosynthesis in ZIKV-infected neuroblastoma cells. **

• In the sentence “ZIKV likely remodels the cellular lipid composition to help produce a favorable environment for efficient replication”, it should be taken into account that ZIKV cannot be the one producing that, but the host viral response be responsible for those effects, and that has not been proved. It could be changed by “ZIKV infection induces all those changes…” 

- **Sentence has been changed to “We propose that lipid abundance, localisation, trafficking and metabolism regulate ZIKV infection of neuroblastoma cells, and that remodelling of the cellular lipid composition within the host cell may produce a favourable environment for efficient replication.” **

# Reviewer report: Reviewer response for version 1

**DOI:** 10.5256/f1000research.145561.r184590

**Published:** 2023-08-17

**Authors:** Griffith D Parks

**Affiliations:** 1 Burnett School of Biological Sciences, University of Central Florida, Orlando, Florida, USA

There is very high interest in mechanisms for improving the potency and specificity of oncolytic viruses, and much of this interest is in large scale analysis of host cell responses. Thus, there is potential significance for the work described here, in the analysis of transcriptomic changes that occur with Zika virus (ZIKV) infection of neuroblastoma cells in culture. An unfortunate weakness is that the work is not presented as what it is: a review of the literature and not a primary study of ZIKV infection.

 Strengths of the work include the importance of understanding host factors that modulate RNA virus infections such as those by ZIKV, the global omics approach to the study using prior data sets and literature searches, the survey of published results to conclude best virus and cell type, and the identification of distinct pathways. The work is largely well done, with interpretations which are consistent with the data.  Figure 7 is particularly useful.

 The main weakness of the manuscript is the presentation of findings as new and “identified” by the authors. For example, for nearly all the sections there are phrases such as “Here, we identify ZIKV NS4B….directly interact with core complexes….” The authors have not identified a direct interaction, but rather have used prior databases to develop a hypothesis that this may occur. This is a misleading representation of the data presented in this manuscript and there should be a re-write of the text to state that this is from analysis of the literature and data mining.

 The authors should make it clear in the abstract in title that this is not a new study, but rather an evaluation of the literature and mining of prior data. In fact, the title is misleading by stating it is a study of ZIKV infection, when it is in reality an analysis of the published data (still useful). In reality, there is a major strength in this large amount of work and the authors are to be commended for it. There needs to be a clear statement that this is a review manuscript.

 Other comments.

1. The text states that ZIKV is neurotropic. This should be modified, as this virus appears to have a lot of different host cell types it can infect. Disease is manifested when there is infection of neuro-cells, but this is not tropism.
2. In the results of abstract – please clarify “ZIKV is dependent on…” What part of ZIKV is dependent?
3. The text does not come to a conclusion on data from Table I other than to say lots of cells can be infected. What is the reason behind the graded difference between the 5 and 4 and 3. The authors speculate on the reason for grade 1, but not the others.
4. Page 4 text – “the Asian lineage…..is the clear choice for oncolytic therapy.” Not clear where this comes from, and no citation was given.
5. Table 2 is useful, but has the caveat that it does not list the cells that were tested. It would be very useful to include a column with “shared cell lines tested in publications”
6. Fig 1D and 2A are entirely too small for anyone to read. These should be expanded and used a separate figures, so as to allow a reader to see the results.

Is the work clearly and accurately presented and does it cite the current literature?

Yes

If applicable, is the statistical analysis and its interpretation appropriate?

I cannot comment. A qualified statistician is required.

Are all the source data underlying the results available to ensure full reproducibility?

Yes

Is the study design appropriate and is the work technically sound?

Yes

Are the conclusions drawn adequately supported by the results?

Partly

Are sufficient details of methods and analysis provided to allow replication by others?

Partly

Reviewer Expertise:

RNA Viruses

I confirm that I have read this submission and believe that I have an appropriate level of expertise to confirm that it is of an acceptable scientific standard, however I have significant reservations, as outlined above.

# Response

**Published:** 2023-11-08

**Authors:** Rob Ewing

**Affiliations:** School of Biological Sciences, University of Southampton, Southampton, England, UK

**We thank Dr Parks for his review of the paper, and his recognition of the significance of identifying mechanisms that may underlie the oncolytic response to the Zika virus in cancer cells. Our intention has been to integrate and re-analyze many of the omics datasets that have studied Zika viral infection of Neuroblastoma cells rather than review the literature. We believe that re-analysis and integration of the available datasets provides many new insights into the Zika virus – neuroblastoma interaction which are not yet available through the papers published to date. Additionally, we believe that re-interpreting and repurposing the non-omics data (infection assays and functional data) from these studies and drawing conclusions on a subject different to that of the original paper (ie ZIKV as an oncolytic therapy rather than as a pathogenic virus infecting neuro-cells) holds great worth. We recognize that we have not in places provided enough clarity of this intention and have now addressed this point throughout the manuscript**. 

 The main weakness of the manuscript is the presentation of findings as new and “identified” by the authors. For example, for nearly all the sections there are phrases such as “Here, we identify ZIKV NS4B….directly interact with core complexes….” The authors have not identified a direct interaction, but rather have used prior databases to develop a hypothesis that this may occur. This is a misleading representation of the data presented in this manuscript and there should be a re-write of the text to state that this is from analysis of the literature and data mining. 

**We have now re-phrased these sections to provide clarification. A representative example is “From our re-analysis of a published ZIKV interactome we propose ZIKV NS4B and NS2B-3 to interact with the core complexes of the mammalian translocon, and hypothesise that ZIKV infection in neuroblastoma cells may be dependent on these interactions”.**

 The authors should make it clear in the abstract in title that this is not a new study, but rather an evaluation of the literature and mining of prior data. In fact, the title is misleading by stating it is a study of ZIKV infection, when it is in reality an analysis of the published data (still useful). In reality, there is a major strength in this large amount of work and the authors are to be commended for it. There needs to be a clear statement that this is a review manuscript. 

**We have now clarified this point throughout the abstract and also through an amended title:**

**“Integrated re-analysis of transcriptomic and proteomic datasets reveals potential mechanisms for Zika viral-based oncolytic therapy in neuroblastoma”**

 Other comments.  

1. The text states that ZIKV is neurotropic. This should be modified, as this virus appears to have a lot of different host cell types it can infect. Disease is manifested when there is infection of neuro-cells, but this is not tropism. 

- **The references to ZIKV being neurotropic have been changed **

1. In the results of abstract – please clarify “ZIKV is dependent on…” What part of ZIKV is dependent? 

- **Has been corrected to “ZIKV infection is dependent on sterol regulatory element binding protein (SREBP)-regulated lipid metabolism and three protein complexes; V-ATPase, ER Membrane Protein Complex (EMC) and mammalian translocon.”.  **

1. The text does not come to a conclusion on data from Table I other than to say lots of cells can be infected. What is the reason behind the graded difference between the 5 and 4 and 3. The authors speculate on the reason for grade 1, but not the others. 

- **We have replaced the rankings with the actual values/ranges to make this easier to interpret.  **
- **We have updated the values of LA-N-6 and SK-N-Be(1). **
- **We have added the following text: “Whilst LA-N-6 shows partial resistance to ZIKV infection ( Table 1), analysis of bulk mRNA and protein show cell LA-N-6 to express CD24. 13 Potential reasoning for this partial resistance is that subpopulations within LA-N-6 may be CD24- or a CD24-independent mechanism may be employed to infer resistance. From Table 1 we conclude ZIKV to be a promising oncolytic virotherapy candidate to employ against paediatric neuroblastoma since it can target neuroblastoma cells originating from the primary tumour, metastatic sites, and metastatic sites that are resistant to standard neuroblastoma therapy.” **

1. Page 4 text – “the Asian lineage…..is the clear choice for oncolytic therapy.” Not clear where this comes from, and no citation was given. 

- **This text has been removed **

1. Table 2 is useful, but has the caveat that it does not list the cells that were tested. It would be very useful to include a column with “shared cell lines tested in publications” 

- **The cell lines have now been added to Table 2, including the reference for the publication from which the data has come from**

1. Fig 1D and 2A are entirely too small for anyone to read. These should be expanded and used a separate figures, so as to allow a reader to see the results.  

- **Both Fig 1D and 2A (the KEGG pathway diagrams) have been pulled out and put into a new figure (Figure 2). Figures have been renumbered accordingly and there are now 8 Figures in total. **
